# Analysis of the Relationship between Scintillation Parameters, Multipath and ROTI

**DOI:** 10.3390/s20102877

**Published:** 2020-05-19

**Authors:** Chendong Li, Craig M. Hancock, Nicholas A. S. Hamm, Sreeja V. Veettil, Chong You

**Affiliations:** 1 Geospatial and Geohazards Research Group, University of Nottingham, Ningbo 315100, China; ssxcl1@nottingham.edu.cn (C.L.); nicholas.hamm@nottingham.edu.cn (N.A.S.H.); 2 Nottingham Geospatial Institute, University of Nottingham, Nottingham NG7 2RD, UK; Sreeja.Veettil@nottingham.ac.uk; 3 Beijing International Center for Mathematical Research, PKU, Beijing 100871, China; Chong.You@nottingham.edu.cn

**Keywords:** GNSS, scintillation parameters, multipath, ROTI, relationship

## Abstract

Global Navigation Satellite System (GNSS) operation can be affected by several environmental factors, of which ionospheric scintillation is one of the most significant. Scintillation is usually characterized by two indices, namely the amplitude scintillation index (S4) and phase scintillation index (σ_φ_). However, these two indices can only be generated by specialized GNSS receivers, which are not widely available all around the world. To popularize the study of scintillation, this article proposes to use more accessible parameters, namely multipath (MP) and rate of change of total electron content index (ROTI), to characterize scintillation. Using GPS data obtained on six days in total from three stations, namely PRU2 and SAO0P located in Sao Paulo, Brazil and SNA0P located in Antarctica, respectively, both the time series plots and 2D maps were generated to investigate the relationship of scintillation indices (S4 and σ_φ_) with MP and ROTI. To prevent the effect of the real multipath error, a 30-degree satellite elevation mask is applied to all the data. As the scintillation indices S4 and σ_φ_ have a sampling interval of 1 min, MP and ROTI are calculated with the same sampling interval for a more direct comparison. The results show that the structural similarity (SSIM) and correlation coefficient (CC) between parameters was greater than 0.7 for 70% of outputs. In addition, the variogram and cross-variogram are applied to investigate the spatial structure of the MP, ROTI, S4 and σ_φ_ in order to support the results of SSIM and CC. With outputs in three forms, promising spatial and temporal relationships between parameters was observed.

## 1. Introduction

Ionospheric scintillation is one of the main error sources that can reduce the quality of positioning outputs or even lead to loss of lock on satellites, and hence can cause significant errors in Global Navigation Satellite System (GNSS) receiver operation [1]. A series of methods have been implemented to mitigate scintillation effects on positioning. For instance, in Aquino et al. [2], the tracking error variances of GNSS receiver phase locked loop (PLL) and delay locked loop (DLL) is used to modify the least squares stochastic model and the positioning accuracy was shown to be improved by 17–21%. Furthermore, Bougard et al. [3] attempted to exclude scintillation affected satellites from the positioning calculation with the technique of receiver autonomous integrity monitoring (RAIM) specific for precise point positioning (PPP), which considerably improved the PPP accuracy. Loss of signal lock and cycle slips are the primary problems under scintillation, which are relatively severe when using a single satellite constellation. Therefore, it was proposed that the data of GPS and GLONASS could be integrated to obtain more reliable positioning solutions under moderate to strong scintillation [4], which improves the accuracy of the positioning output by 60% compared to using GPS alone. By using these approaches, the effects of scintillation can be mitigated to a certain extent. However, specialized receivers that can generate the scintillation indices, namely the amplitude scintillation index (S4) and phase scintillation index (σ_φ_) [5] are not available worldwide, which limits the application of these approaches. Though the S4 index can be computed with low-cost receivers or common geodetic GNSS receivers [6,7], the σ_φ_ index cannot be generated in the same way. In order to overcome this problem, it is proposed to investigate the relationship of two parameters, namely multipath (MP) and rate of change of total electron content index (ROTI), that can be obtained from standard generic receivers to represent scintillation indices (S4 and σ_φ_).

Studies on the relationship between scintillation and MP or ROTI were initiated respectively by Romano et al. [8] and Basu et al. [9]. According to Romano [8], a certain relationship can be observed between MP and scintillation parameters. Specifically, MP has a negative influence on the presentation of ionospheric scintillation [8]. Hancock et al. [10] also demonstrated an agreement between MP and scintillation parameters. It has been demonstrated previously that ROTI can be used to indicate the occurrence of scintillation [9]. It has been further demonstrated that the correlation between ROTI and scintillation parameters is strong with a correlation coefficient exceeding 0.6 on average, even higher than 0.8 sometimes [11]. In this paper, the relationship between MP and ROTI with S4 and σ_φ_ in both the spatial and temporal domains is investigated. Additionally, propagation patterns of all four parameters are studied to investigate the spatial similarity over time.

The purpose of this study is to investigate the relationship of S4 and σ_φ_ with MP and ROTI. Several objectives are set as follows:

(1) Compare the time series plots of the four parameters to observe the temporal relationship to confirm if during periods of scintillation all parameters are similarly affected.
(2) Thereafter, two types of 2D maps are constructed, mean value maps and occurrence percentage maps, where the former is to evaluate whether abnormally high value areas are in similar spatial regions and the latter is to investigate the referred areas with clearer outputs. As scintillation mainly occurs at night and in the early morning local time, the maps are first generated with a period of 6 h.
(3) Furthermore, the maps with 5 min periods are generated to observe the relationship during times when the largest variations of the parameters are observed.
(4) Finally, the structural similarity (SSIMs) and Pearson correlation coefficient (CC) between maps are calculated to evaluate the similarity between the parameters. Variograms and cross-variograms are also used to evaluate the spatial correlation in the maps.

## 2. Materials and Methods

### 2.1. Data and Instrumentation

Data used in this study were collected from three stations, PRU2 (51.41° W, 22.12° S), SAO0P (46.65° W, 23.55° S) located in Sao Paulo, Brazil and SNA0P (2.84° W, 71.67° S) located in Antarctica. The receivers in PRU2, SAO0P and SNA0P stations are Septentrio PolaRxS 2.1.1, Septentrio PolaRxS 2.9.6 located in Sao Paulo, Brazil and Septentrio PolaRxS 2.9.0 located in Antarctica, respectively. The Septentrio PolaRxS is a multi-frequency multi-constellation receiver specialized for monitoring ionospheric activity. As scintillation activity is mainly strong in two global areas, namely the equatorial and polar regions, where the causes leading to the generation of scintillation are completely different [12], data from three stations located in these two regions is used. All the parameters are acquired with a sampling interval of 1 min for the purpose of comparison.

### 2.2. Software

MATLAB version 2019a and TEQC [13,14] were the main software used in this study. MATLAB was used for calculation and visualization, whereas TEQC was utilized to generate quality control (QC) parameters. The R Language and Environment for Statistical Computing [15] (Version 3.5.1) and the R package gstat (Version 1.1–6) [15,16,17] was used for the geostatistical computations.

### 2.3. ROTI

ROTI is one of the two standard parameters that is calculated. The Total Electron Content (TEC) is obtained first, according to Holding [18]:

(1) $$P_{i}=\rho +c\left(\delta_{R}-\delta^{S}\right)+c\left(B_{R}-b^{S}\right)+I_{i}+T+M_{PR_{i}}+\varepsilon$$

where P is the geometric distance including errors; i is 1 or 2, representing two frequencies; ρ is the true geometric distance between the satellite and receiver; c is the speed of the light; $\delta_{R}$ is the receiver clock error; $\delta^{S}$ is the satellite clock error; $B_{R}$ is code bias caused by receiver hardware delay; $b^{S}$ is code biases caused by satellite hardware delay; I is the ionospheric delay; T is the tropospheric delay; $M_{PR}$ is the multipath error (i.e., multipath pseudo range error); and ε represents other unconsidered errors. Then raw TEC values can be calculated with Equations (2)–(4):

(2) $$\frac{I_{1}}{I_{2}}=\frac{f_{2}^{2}}{f_{1}^{2}}$$

(3) $$P_{1}-P_{2}=I_{1}-\frac{f_{1}^{2}}{f_{2}^{2}}I_{1}$$

(4) $$STEC=\frac{1}{40.3}\left(\frac{f_{1}^{2}f_{2}^{2}}{f_{1}^{2}-f_{2}^{2}}\right)\left(P_{2}-P_{1}\right)$$

where f is the frequency of the GNSS signal and the subscript i is as mentioned above. STEC is the slant TEC representing the TEC along the satellite-receiver link. STEC can be converted into vertical TEC (VTEC) using the mapping function defined in [18] as:

(5) $$\mathrm{VTEC}=\mathrm{STEC}\cos\left(z^{\prime }\right)=\mathrm{STEC}\sqrt{1-\sin^{2}\left(z^{\prime }\right))}$$

(6) $$\sin z^{\prime }=\frac{R}{R+H}\;\sin z$$

where R is radius of the Earth and H is the height of the ionosphere layer. ROTI is defined as the standard deviation of the rate of VTEC (ROT) as in [19]:

(7) $${\mathrm{ROT}}_{t_{n}}=\frac{{\mathrm{VTEC}}_{t_{n}}-{\mathrm{VTEC}}_{t_{n-1}}}{t_{n}-t_{n-1}}$$

(8) $$\mathrm{ROTI}=\sqrt{\langle {\mathrm{ROT}}^{2}\rangle -{\langle \mathrm{ROT}\rangle }^{2}}$$

In this study, the ROTI estimated over a 5-min interval is used, which was originally defined by Pi et al. [19]. Additionally, the moving average is applied in ROTI calculation so that the time interval is the same as other parameters.

### 2.4. MP

Multipath is a type of interference to GNSS receivers which is caused by reflected signals. In this paper, a 30° satellite elevation mask is applied to all the parameters, which removes most of the multipath effects. An assumption is then made that in general, MP values that are higher than average for signals above 30° during scintillation events are likely to be affected by scintillation and not multipath. MP1 and MP2 can be calculated using Equations (9) and (10) defined in [13]:

(9) $$\begin{array}{c} MP1\equiv P_{1}-\left(1+\frac{2}{a-1}\right)L_{1}+\left(\frac{2}{a-1}\right)L_{2}\\ \;\;\;\;\;\;\;=M_{PR_{1}}+B_{1}-\left(1+\frac{2}{a-1}\right)M_{CP_{1}}+\left(\frac{2}{a-1}\right)M_{CP_{2}} \end{array}$$

(10) $$\begin{array}{c} MP2\equiv P_{2}-\left(\frac{2a}{a-1}\right)L_{1}+\left(\frac{2a}{a-1}-1\right)L_{2}\\ \;\;\;\;\;\;=M_{PR_{2}}+B_{2}-\left(\frac{2a}{a-1}\right)M_{CP_{1}}+\left(\frac{2a}{a-1}-1\right)M_{CP_{2}} \end{array}$$

where $P_{i}$ is the pseudorange observable for frequency i; $L_{i}$ is the phase observable for frequency i; $M_{CP_{i}}$ is the carrier phase multipath for frequency i; $a=\frac{f_{1}^{2}}{f_{2}^{2}}$, where $f_{i}$ is the frequency of signal i; $B_{i}$ is a bias terms, which is defined as:

(11) $$B_{1}\equiv -\left(1+\frac{2}{a-1}\right)n_{1}\lambda_{1}+\left(\frac{2}{a-1}\right)n_{2}\lambda_{2}$$

(12) $$B_{2}\equiv -\left(\frac{2a}{a-1}\right)n_{1}\lambda_{1}+\left(\frac{2a}{a-1}-1\right)n_{2}\lambda_{2}$$

where $n_{i}\lambda_{i}$ is the integer wavelength phase ambiguity for frequency i. In this paper, MP1 and MP2 are generated using the quality control (QC) command of the TEQC software on the receiver independent exchange format (RINEX) version 2.11 files from the three stations, which include both observation and navigation files [14].

### 2.5. Ionospheric Pierce Point (IPP)

As this study focuses on ionospheric effects, the results are analyzed at the ionospheric boundary, where the IPP coordinates are calculated. Prior to that, an intermediate quantity, which is the intersection angle of receiver and the IPP to the center of Earth, is calculated using Equation (13):

(13) $$p=\frac{\pi }{2}-ele-\sin^{-1}\left(\frac{Rcos\left(ele\right)}{R+H}\right)$$

where ele is the elevation angle of the satellite from the receiver. The coordinates of the IPP can then be calculated in latitude and longitude using Equations (14) and (15):

(14) $$\phi_{IPP}=\sin^{-1}\left(\sin\left(\phi \right)\cos\left(p\right)+\cos\left(\phi \right)\sin\left(p\right)\cos\left(azi\right)\right)$$

(15) $$\lambda_{IPP}=\lambda +\sin^{-1}\left(\sin\left(p\right)\sin\left(azi\right)/\cos\left(\phi \right)\right)$$

where ϕ and λ are respectively the latitude and longitude of the receiver position; azi is the azimuth angle of the satellite from the receiver.

### 2.6. Methodology

**Figure 1:**
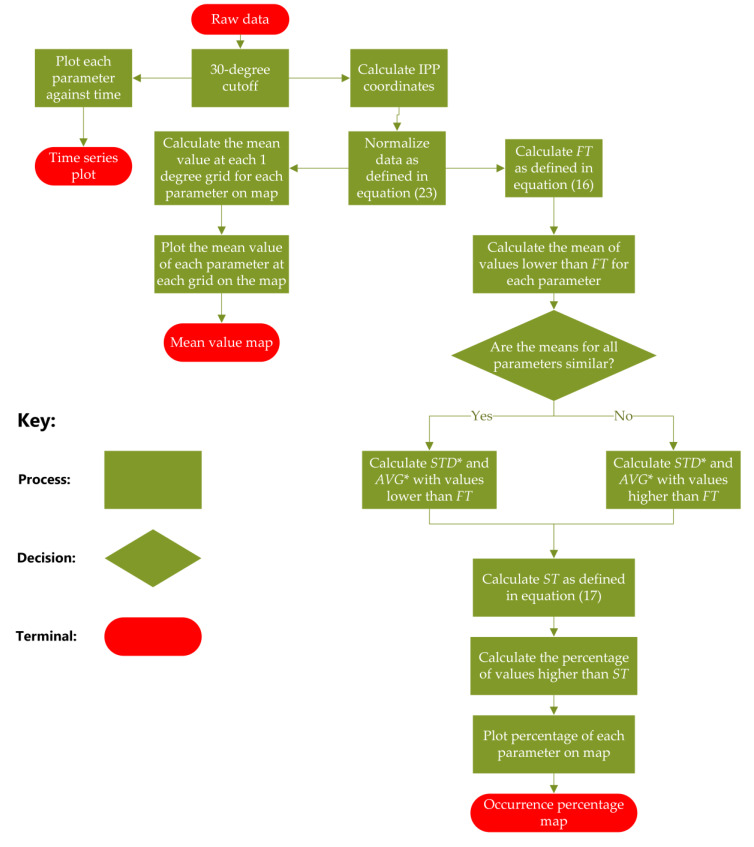
Flow chart for data processing and visualization.

Figure 1 shows the flowchart of the methodology. As shown in Figure 1, 30° cutoff is applied to all the data. The time series plots of all four parameters are generated before normalization to observe the general relationship and the scintillation intensity. Then all the data is normalized using the normalization method namely P-Norm [20] as described in Section 2.9 so that the different parameters vary in the same range. Next, two types of maps are generated, the mean value map and the occurrence percentage map, where each map is generated using data from a single station with a spatial resolution of 1°. Mean value maps are generated first to investigate the spatial relationship, where the mean values are calculated for each grid cell. To achieve this the parameter values with the IPP coordinates located in the same grid are averaged and visualized on a map. After that, in order to more clearly visualize high values, occurrence percentage maps are generated. These show the percentage of values greater than a threshold for each 1° grid cell. The method for calculating the threshold is given in Equation (17). Compared with the mean value map, this map shows only the extreme values.

To generate these extreme values a threshold is applied, which is determined using the standard deviation and mean of values for each parameter. For the Antarctic data, as the station is located in the high latitudes, phase scintillation is considerably more likely than amplitude scintillation [12,21]. Therefore, S4 is not considered for the Antarctic data, where MP, ROTI and σ_φ_ fluctuate with different magnitudes from each other during the period with low scintillation and with similar magnitudes to each other during scintillation period. In order to mitigate biases, the different parameters values should be filtered first, where the values lower than a threshold as defined in Equation (17) are not used for occurrence percentage calculation.

This is realized by applying two thresholds, determined using Equations (16) and (17) below:

(16) FT=STD+AVG

(17) $$ST=x\cdot STD^{*}+AVG^{*}$$

where FT and ST respectively represent the first threshold and the second threshold; STD and AVG are the standard deviation and mean of all values for each parameter; $STD^{*}$ and $AVG^{*}$ are the standard deviation and mean of values higher or lower than FT, dependent on the characteristics of the data, which is explained in more detail in Figure 1; x is a real number, which can be adjusted in order to distinctly show maps within different durations. For the Antarctic data, the size of the four parameters during the non-scintillation period are of different magnitude. Take the dataset in Antarctica on 2 April 2017 as an example, the mean of MP, ROTI, S4 and σ_φ_ during non-scintillation period (values lower than FT) respectively are 0.00026, 0.0032, 0.00051, 0.00066, where ROTI is not of the same magnitude as the other three parameters. Therefore, values higher than the FT is used for the ST calculation. For the Brazilian data, the situation is different, the sizes of all four parameters during non-scintillation period vary with similar magnitude to each other. Take the dataset in Brazil on 13 September 2017 as an example, the mean of MP, ROTI, S4 and σ_φ_ during non-scintillation period (values lower than FT) respectively are 0.00033, 0.00045, 0.00030 and 0.00057, which are similar to each other. Hence, the values lower than the FT are used for calculating ST for the Brazilian data. After following this process, the percentage of number of values larger than the thresholds can be drawn on each grid of maps. In this paper, thresholds are chosen to be relatively large (x is comparatively large) in order to show distinct extreme value areas on percentage occurrence maps.

In addition to generating mean value maps for 6 h, maps were also constructed for 5-min duration during scintillation period. Maps containing data for five minutes with scintillation are generated in order to compare parameters at more specific time.

### 2.7. Structural Similarity (SSIM) and Pearson Correlation Coefficient (CC)

The structural similarity index (SSIM) is an index for quantifying the similarity between two images. Three aspects are combined: luminance, contrast and structure [22]. These three components can show different characteristics of images. The comparison of luminance (l(m,n)), contrast (c(m,n)) show the difference in the means and variances respectively whereas structure (s(m,n)) quantifies the correlation. The similarities between images are higher with larger SSIM values. Therefore, SSIM is applied here for comparing maps of scintillation indices with those of MP and ROTI in order to describe spatial similarities. SSIM is calculated according to Equations (18)–(20) [22]:

(18) $$\begin{array}{c} l\left(m,n\right)=\frac{2\mu_{m}\mu_{n}+C_{1}}{\mu_{m}^{2}+\mu_{n}^{2}+C_{1}},\\ c\left(m,n\right)=\frac{2\sigma_{m}\sigma_{n}+C_{2}}{\sigma_{m}^{2}+\sigma_{n}^{2}+C_{2}},\\ s\left(m,n\right)=\frac{\sigma_{mn}+C_{3}}{\sigma_{m}\sigma_{n}+C_{3}} \end{array}$$

(19) SSIM(m,n)=l(m,n)×c(m,n)×s(m,n)

(20) $$\begin{array}{c} C_{1}={\left(K_{1}L\right)}^{2},\\ C_{2}={\left(K_{2}L\right)}^{2},\\ C_{3}=C_{2}/2 \end{array}$$

where m and n are two non-negative image signals, $\mu_{m}$ is the mean of image m, $\mu_{n}$ is the mean of image n, $\sigma_{m}^{2}$ is the variance of image m, $\sigma_{n}^{2}$ is the variance of image n, $\sigma_{mn}$ is the covariance between image m and n. The parameters $C_{1}$ and $C_{2}$ were introduced to prevent instability if the value of the denominator is small. Following convention $K_{1}$ = 0.01, $K_{2}=0.03$ and *L* is the dynamic range of the pixel values [22]. In this research, the default SSIM command in MATLAB version 2019a was used.

The correlation between m and n is defined as $\sigma_{mn}/\sigma_{m}\sigma_{n}$. This is estimated using the standard Pearson correlation coefficient (CC). Note from Equation (18) that this is the same as s(m, n) except for the inclusion of $C_{3}$. CC and s(m, n) can differ substantially when CC is small. This can also lead to high values of SSIM.

### 2.8. Variograms

**Figure 2:**
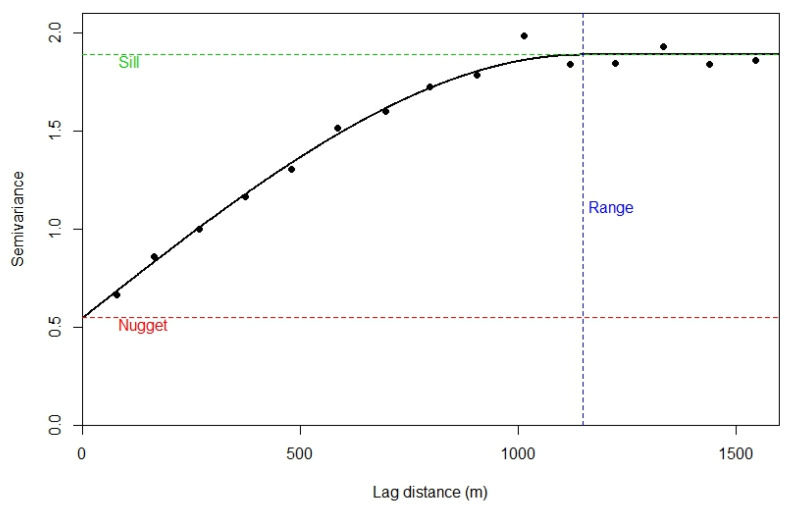
Illustration of the sample variogram (black points) and modelled variogram (black line). This is for an example dataset of heavy metals from the River Meuse in the Netherlands (Pebesma, 2004).

Geostatistics provides a set of tools and methods to analyze spatially referenced data. Of particular interest is a tool known as the sample variogram, which is defined as:

(21) $$\hat{\gamma }\left(h\right)=\frac{1}{n\left(h\right)}\;\overset{n\left(h\right)}{\underset{k=1}{\sum }}{\left(y\left(u_{k}\right)-y\left(u_{k}+h\right)\right)}^{2}$$

where $\hat{\gamma }\left(h\right)$ is the semi-variance for two points separated by distance h, y(u) is the attribute value at location u and n(h) is the number of observations separated by distance h. The variogram provides information about the spatial dependence and spatial structure in the data. When $\hat{\gamma }\left(h\right)$ is small, the difference between two observations of y, separated by h is expected to be small. When $\hat{\gamma }\left(h\right)$ is large, the difference is expected to be large. An example variogram for field observations of the heavy metal, Cadmium, is shown in Figure 2.

The solid line in Figure 2 is a model that has been fitted to the sample variogram. This allows us to estimate γ(h) for all values of h. The model is parameterized by the sill, nugget and range. The variogram sill represents the maximum variability in the data. The nugget represents a combination of non-spatial variability and micro-scale variability (variability at lags less than min(h)). The range is the limit of spatial correlation. We expect two observations separated by distances larger than the range to be uncorrelated. A flat variogram (commonly referred to as “pure nugget”) indicates that there is no spatial structure in the data.

The concept behind the variogram can be extended to consider the spatial cross-correlation between two variables. This allows us to investigate how correlated $y_{1}\left(u\right)$ is with $y_{2}\left(u+h\right)$. The sample cross-variogram is given as:

(22) $$\gamma_{yz}\left(h\right)=\frac{1}{n\left(h\right)}\;\overset{n\left(h\right)}{\underset{i=1}{\sum }}\left(y_{1}\left(u_{k}\right)-y_{1}\left(u_{k}+h\right)\right)\left(y_{2}\left(u_{k}\right)-y_{2}\left(u_{k}+h\right)\right)$$

The sample variogram and modelled variogram can be used as exploratory tools to investigate the spatial structure in a dataset. They can also be used in predictive mapping to interpolate datasets. The concepts are further explained in detail by Goovaerts et al. [23], Webster and Oliver [24] and Webster et al. [25]. Cressie [26] gives a more detailed theoretical treatment. Examples in remote sensing are given by Atkinson and Curran [27], Van der Meer [28] and Odongo et al. [29].

Here we use the variogram and cross-variogram to investigate the spatial structure in the four variables (MP, ROTI, S4, σ_φ_) and to investigate the cross-correlation between the four variables. In all calculations we used degrees as the unit of distance. We acknowledge that it is preferred to work with a projected coordinate system. However, we defend this choice because we work with reasonably small distances and use the variogram for data exploration rather than for prediction.

### 2.9. Normalization

As various parameters vary greatly in their maximum and minimum values, normalization is applied so that parameters can be compared within a certain size range. P-Norm is applied in this research, which is defined by MATHWORKS [20] as Equations (23) and (24):

(23) $${\left\|v\right\|}_{p}={\left[\overset{N}{\underset{l=1}{\sum }}{\left|v_{l}\right|}^{q}\right]}^{1/q}$$

(24) $$v_{n}=\frac{v_{l}}{{\left\|v\right\|}_{p}}$$

where ${\left\|v\right\|}_{p}$ is P-Norm; $v_{l}$ is the vector before normalization; l is the serial number of $v_{l}$; N is the total number of vectors; q can be any positive real value, which is selected to be 2 here namely 2-Norm because it is the standard norm and most widely applied in P-Norm; $v_{n}$ is the normalized vector.

## 3. Results

### 3.1. Brazil Data

**Figure 3:**
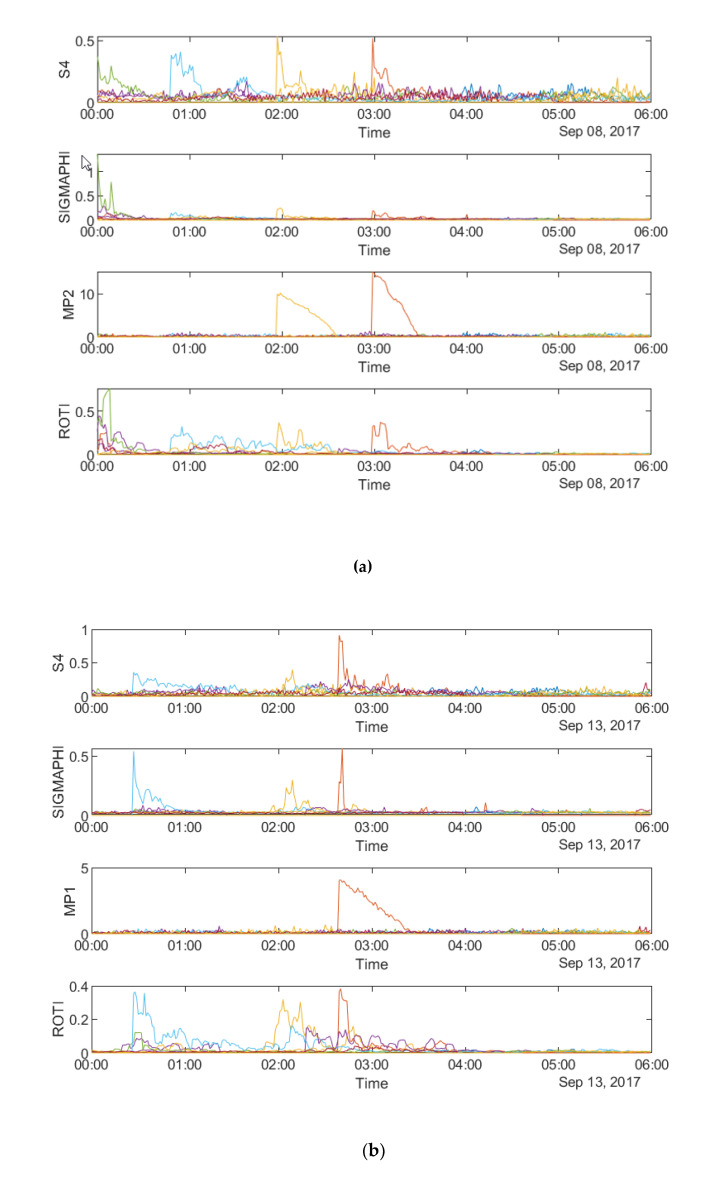

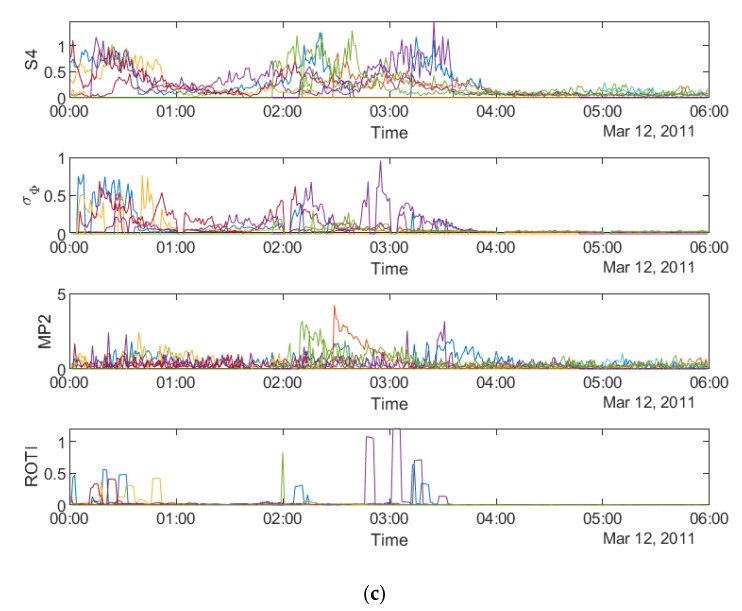
Time series plots of four parameters: (**a**) during 00:00:00–06:00:00 UTC on 8 September, 2017 at SAO0P station; and (**b**) during 00:00:00-06:00:00 UTC on 13 September 2017 at SAO0P station; (**c**) during 00:00:00–06:00:00 UTC on 12 March 2011 at PRU2 station.

Figure 3a–c shows the time series of the four parameters, namely S4 (top row), σ_φ_ (second row from top), MP (third row from top) and ROTI (bottom row) respectively on 8 September 2017 and 13 September 2017 at SAO0P station and 12 March 2011 at PRU2 station.

**Figure 4:**
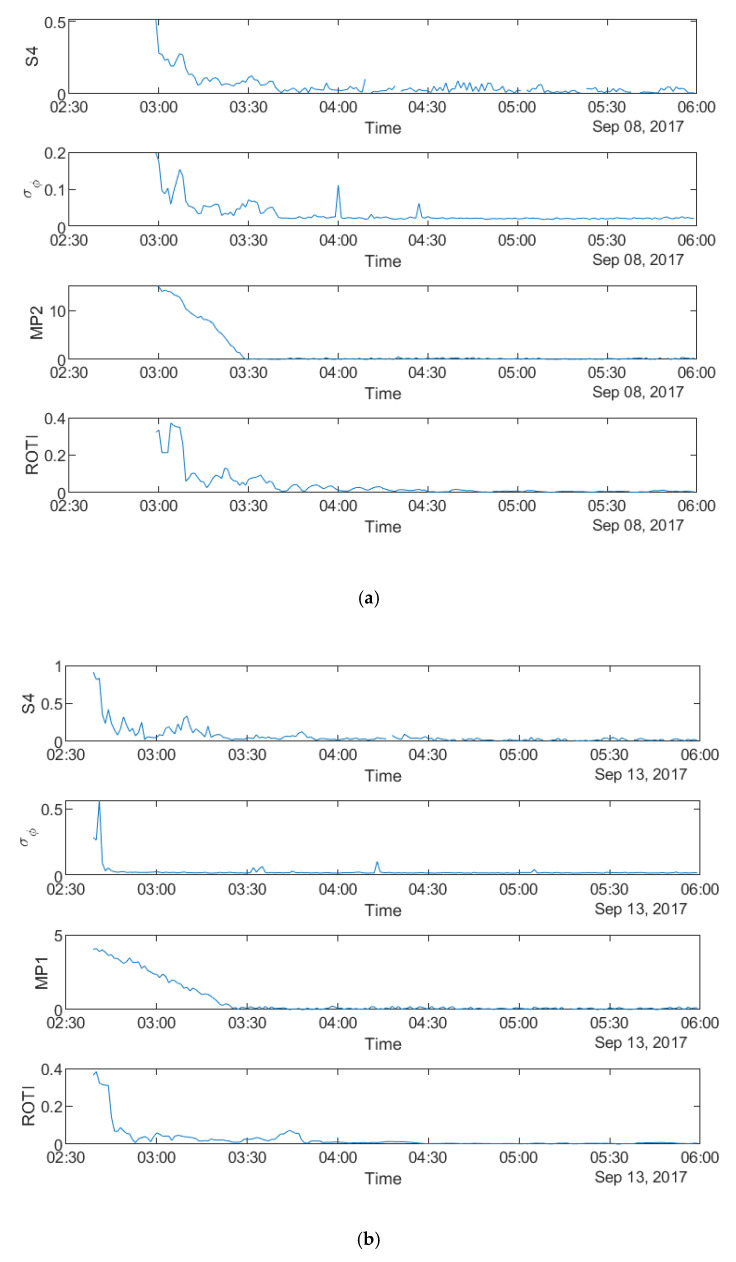

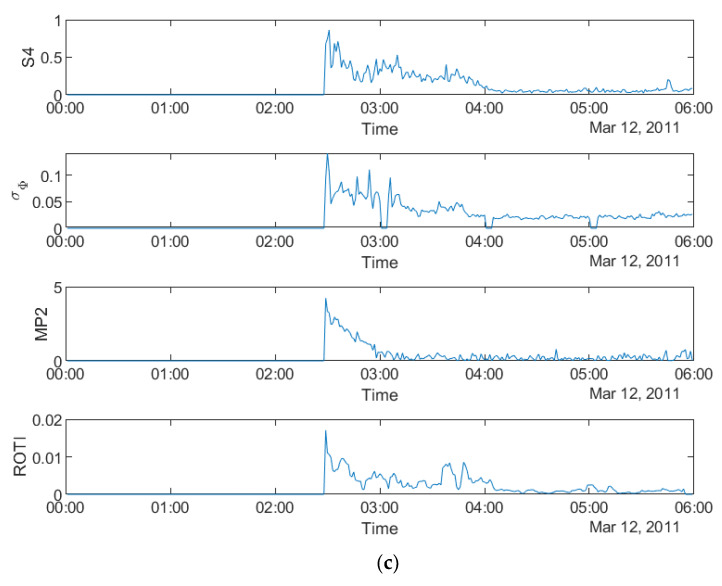
Time series plots of four parameters: (**a**) during 00:00:00-06:00:00 UTC on 8 September 2017 for PRN10 at SAO0P station; (**b**) during 00:00:00–06:00:00 UTC on 13 September 2017 for PRN10 at SAO0P station; (**c**) during 00:00:00–06:00:00 UTC on 12 March 2011 for PRN25 at PRU2 station.

Figure 3 shows that all four parameters fluctuate during the first four hours on all the three days, which indicates a relationship between the parameters in the time domain. It can be seen from all the panels that there are fewer peaks on the MP plots which means that fewer satellites are affected by MP than scintillation. Figure 4a–c shows the time series plots of four parameters for a single satellite respectively on 8 September 2017 and 13 September 2017 at SAO0P station and 12 March 2011 at PRU2 station. As shown in Figure 4, the temporal relationship becomes clearer with the time series plots for a single satellite, where all four parameters have a similar peak period on each day.

**Figure 5:**
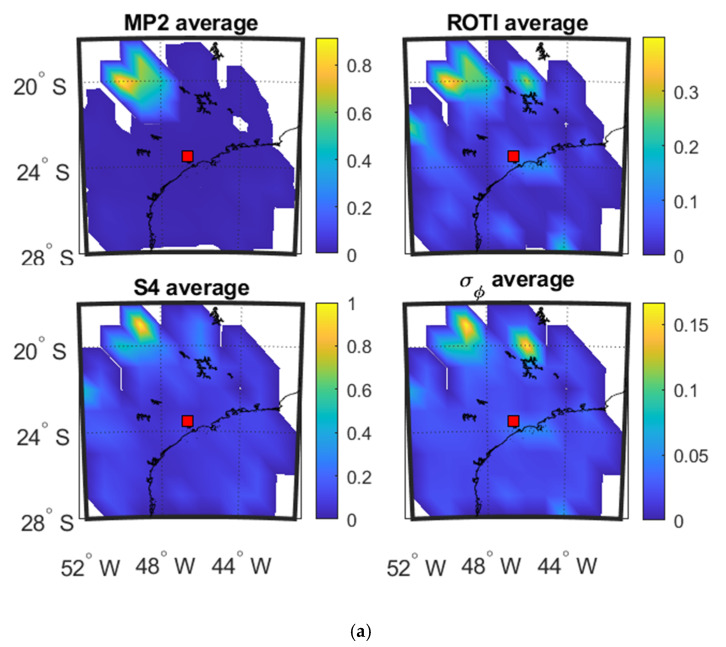

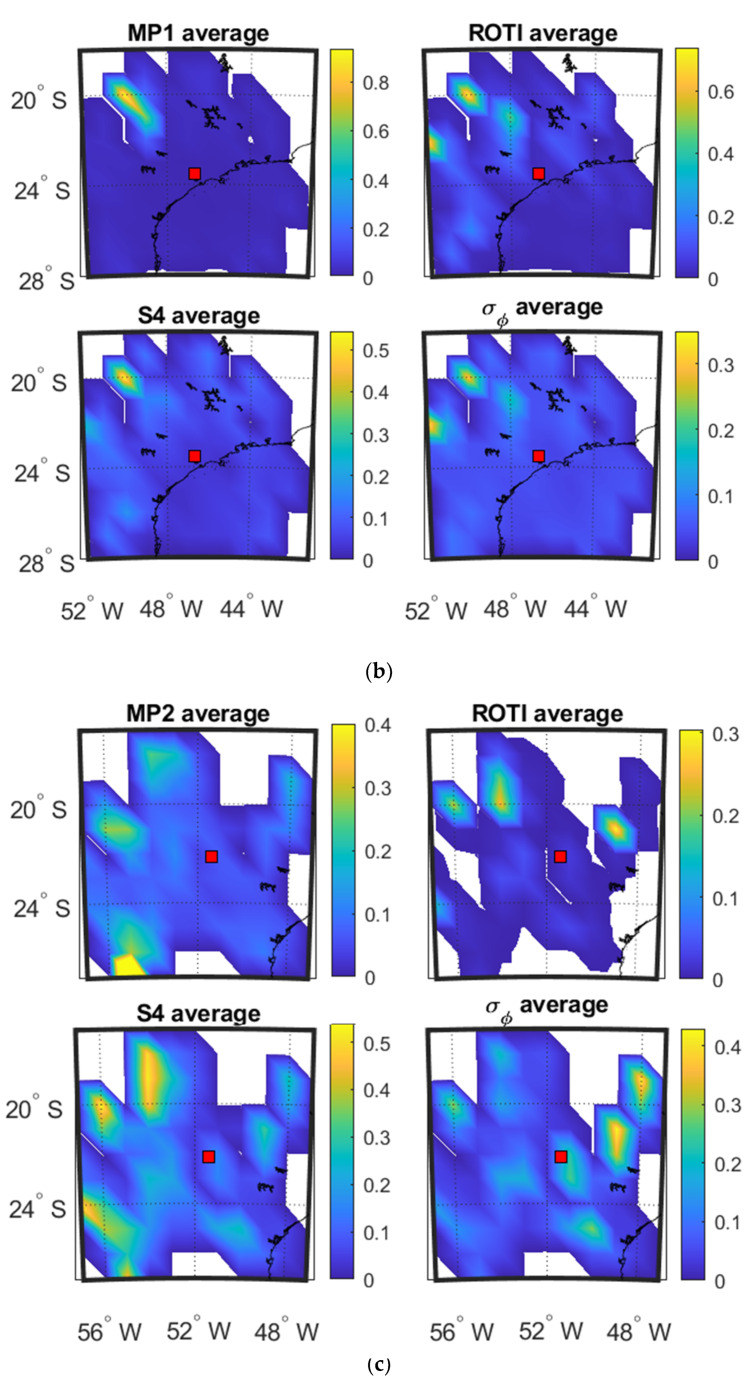
Mean value maps of four parameters: (**a**) during 00:00:00–06:00:00 UTC on 8 September 2017 at SAO0P station; and (**b**) during 00:00:00–06:00:00 UTC on 13 September 2017 at SAO0P station; (**c**) during 00:00:00-06:00:00 UTC on 12 March 2011 at PRU2 station.

The panels a–c of Figure 5 show the spatial mean value 2D maps as a function of IPP latitude and longitude calculated in each 1° grid cell, for the four parameters respectively on 8 September and 13 September 2017 at SAO0P station and 12 March 2011 at PRU2 station.

It can be observed from Figure 5a,b that the affected areas (shown in yellow) of all the parameters focus on the northwest corner on the maps, which indicates that there is a relationship between the parameters in the spatial domain. As shown in Figure 5c, high value areas of S4 and MP are both located on the left part of maps while ROTI agrees with σ_φ_ on the east part of maps, though ROTI also shows similarities in the northwest corner with S4. The same relationship pattern can also be observed from Figure 5a,b that MP mainly correlates with S4 while ROTI respectively correlates with part of S4 and σ_φ_.

Table 1, Table 2 and Table 3 show the SSIM and CC calculated between the 4 parameters shown in Figure 5a–c respectively. From Table 1, Table 2 and Table 3, it is clear that MP is more correlated with S4 while ROTI is more highly correlated with σ_φ_, although the CC in Table 3 shows a different pattern of ROTI. Most of the SSIM and CC values exceed 0.6 and some exceed 0.8. This indicates a high correlation between the parameters and a high level of structural similarity.

**Figure 6:**
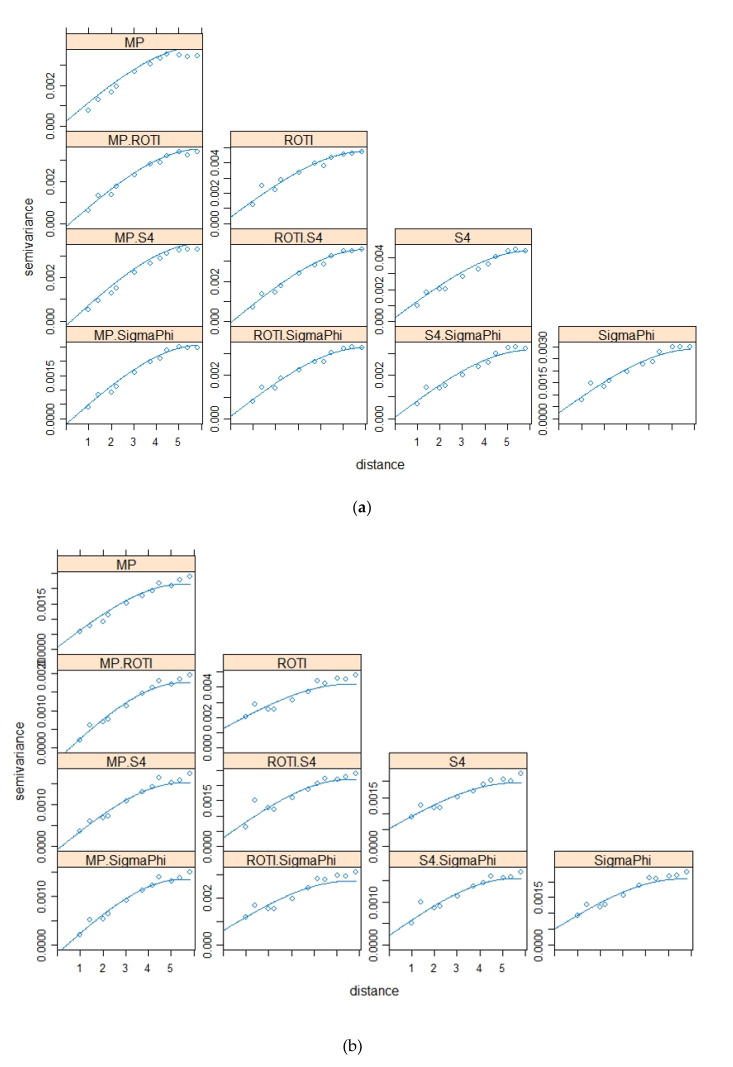

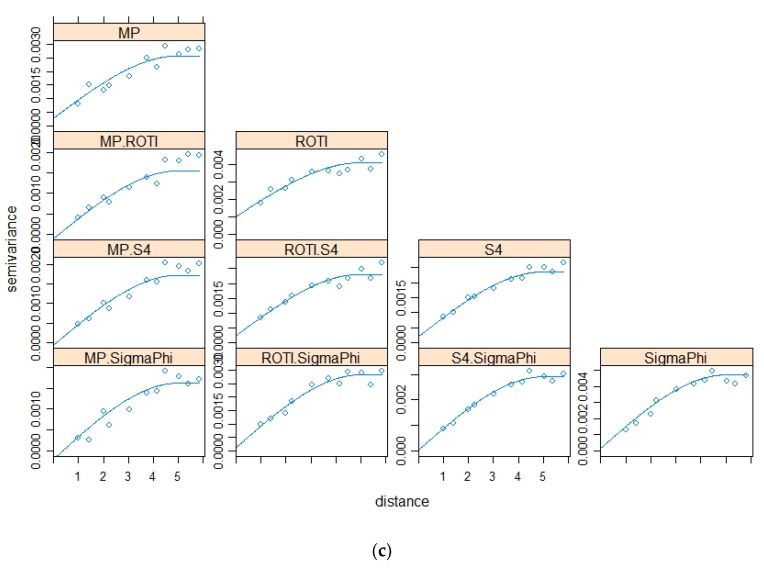
Variograms and cross-variograms for the four parameters. Figure 6a–c is for the data illustrated in Figure 5a–c.

The variograms and cross variograms for Figure 5a,b,c are shown in Figure 6a,b,c respectively. The variogram and cross-variograms show clear evidence of spatial structure, with a range of approximately 5° to 6°. This shows that the four variables (parameters) co-vary, suggesting that they have similar spatial structures.

**Figure 7:**
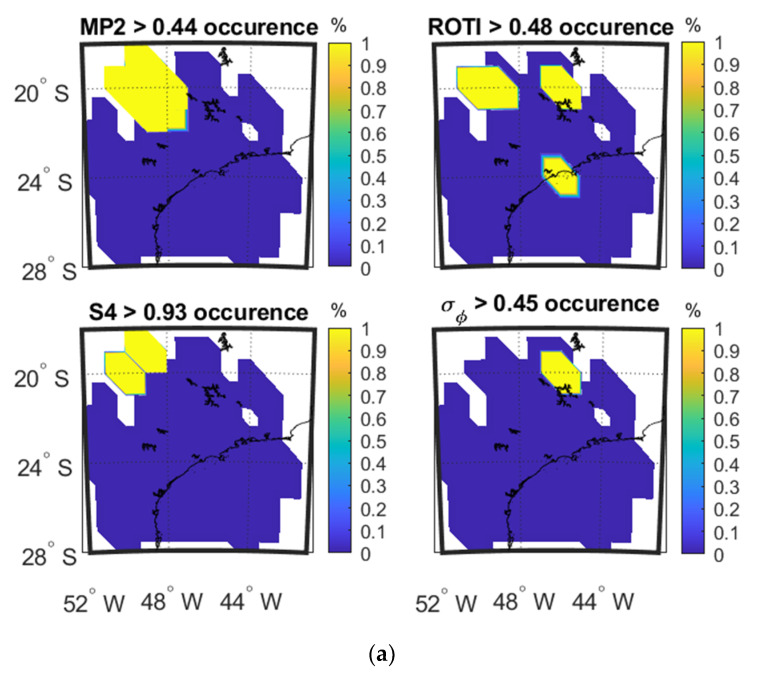

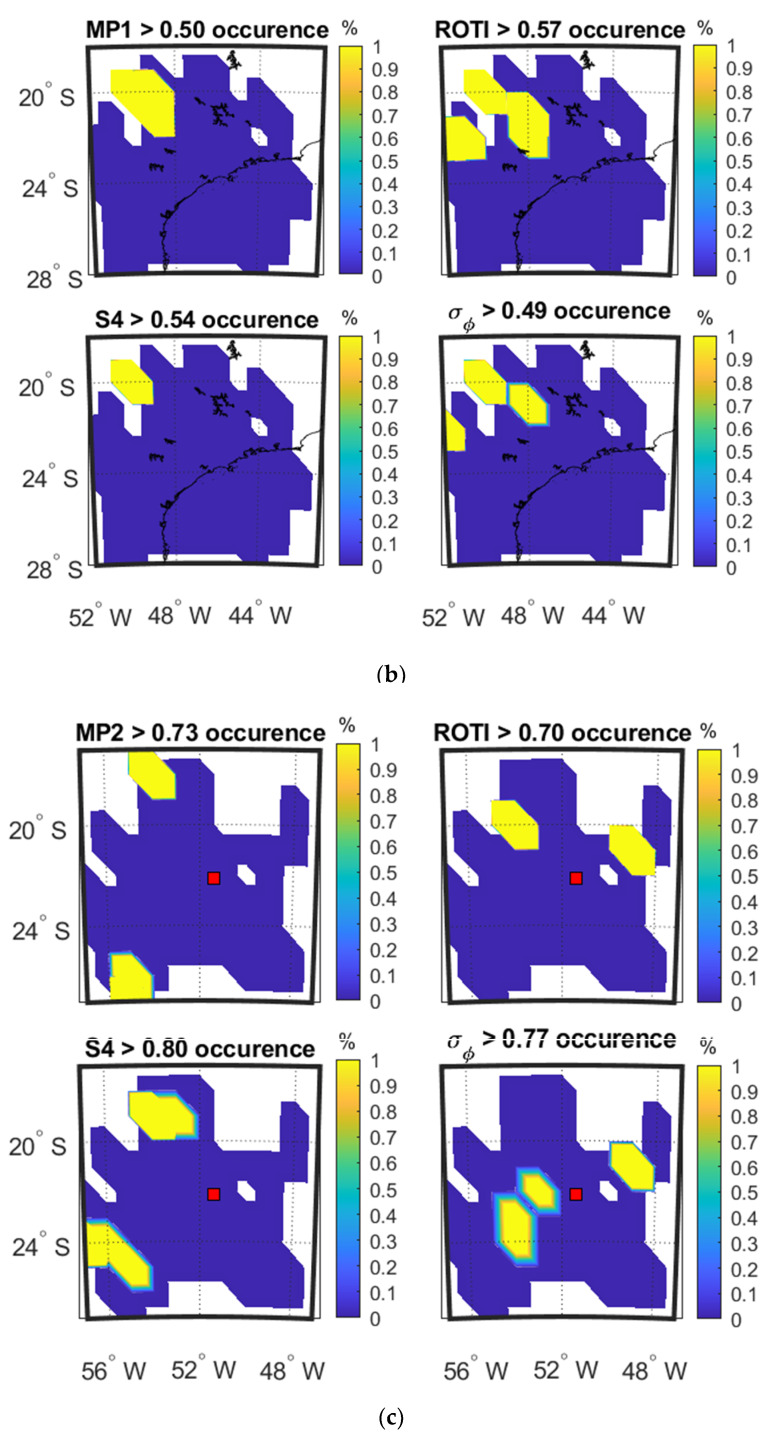
Occurrence percentage maps of four parameters: (**a**) during 00:00:00–06:00:00 UTC on 8 September, 2017 at SAO0P station; (**b**) during 00:00:00–06:00:00 UTC on 13 September 2017 at SAO0P station; (**c**) during 00:00:00–06:00:00 UTC on 12 March 2011 at PRU2 station.

Figure 7a–c show the occurrence percentage maps as a function of IPP latitude and longitude for the four parameters on 8 September and 13 September 2017 at SAO0P station and 12 March 2011 at PRU2 station.

Figure 7a shows that high values of MP cover the high values of S4 while high values of ROTI cover high value of σ_φ_. A similar result is observed in Figure 7b. For Figure 7c, high values of MP do not clearly overlap with high values of S4 and high values of ROTI do not clearly overlap with high values of σ_φ_. These observations are reflected in the CC values shown in Table 4, Table 5 and Table 6.

**Figure 8:**
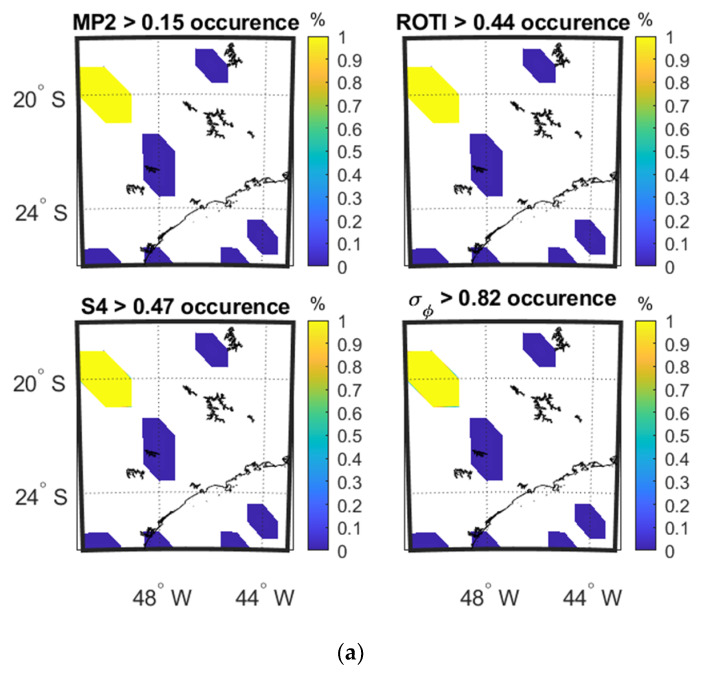

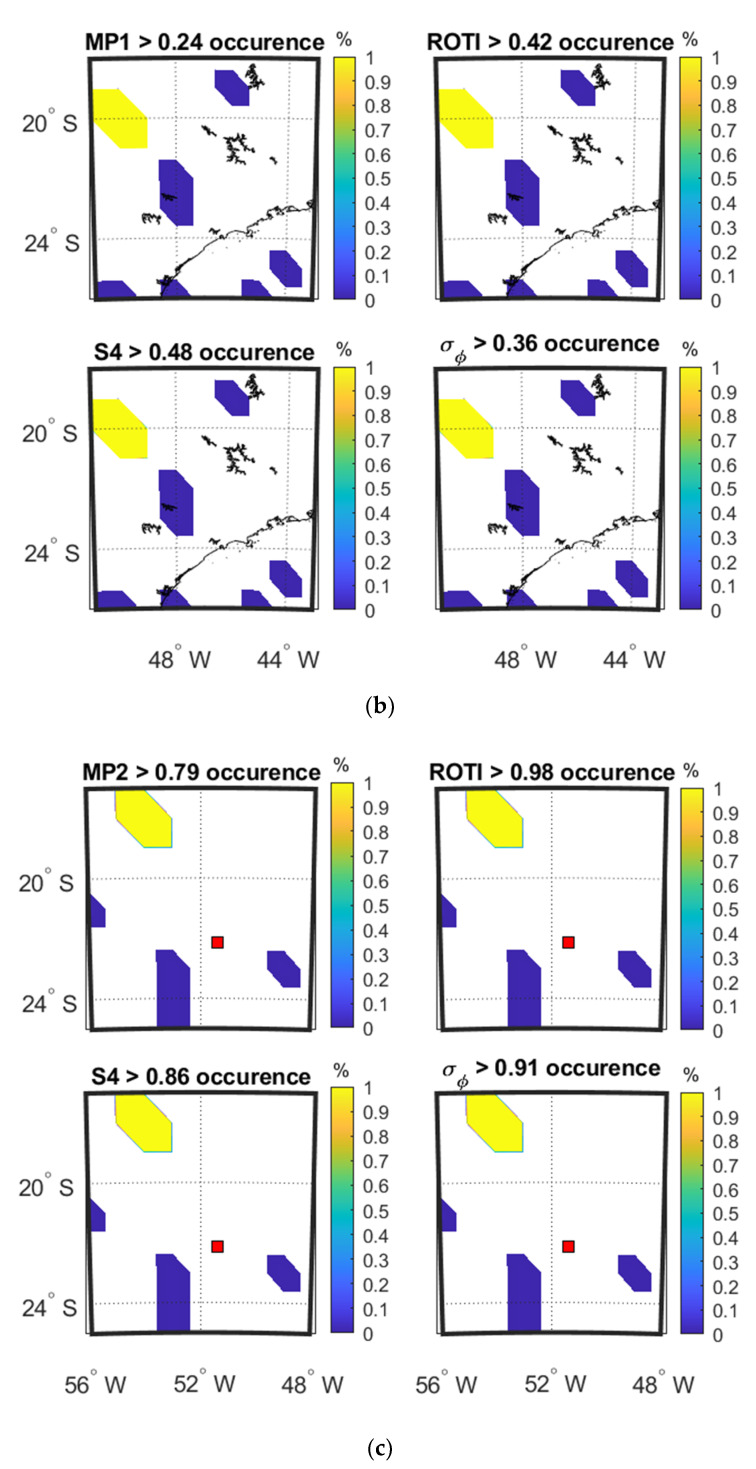
Occurrence percentage maps of four parameters: (**a**) during 02:59:00–03:04:00 UTC on 8 September 2017 at SAO0P station; (**b**) during 02:39:00–02:44:00 UTC on 13 September 2017 at SAO0P statinon; (**c**) during 03:31:00–03:36:00 UTC on 12 March 2011 at PRU2 station.

Figure 8a–c shows the occurrence percentage maps as a function of IPP latitude and longitude of four parameters on 8 September and 13 September 2017 at SAO0P station and 12 March 2011 at PRU2 station during the period when the parameters show the largest variation as observed from Figure 3.

Taking a data period of 5 min with scintillation, as shown in Figure 8, all the parameters are active at the same locations. The SSIM and CC between the parameters in the maps of Figure 8a,b,c are shown in Table 7, Table 8 and Table 9 respectively. These show that the four parameters are perfectly correlated in all four maps. The SSIMs are less than 1 in Table 7 and Table 8, indicating that the mean values of the parameters differ.

**Figure 9:**
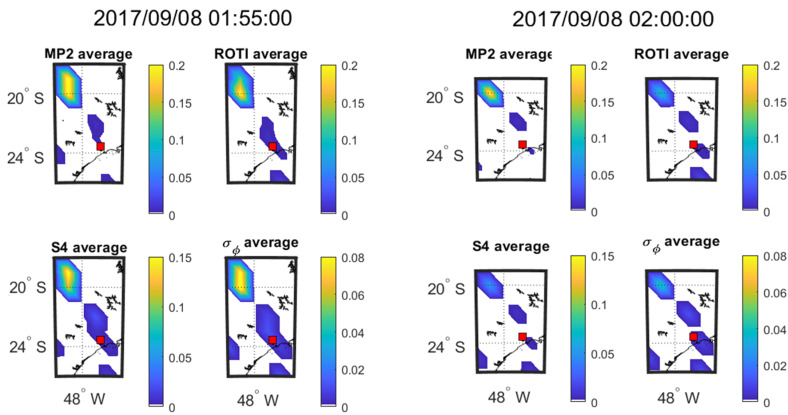

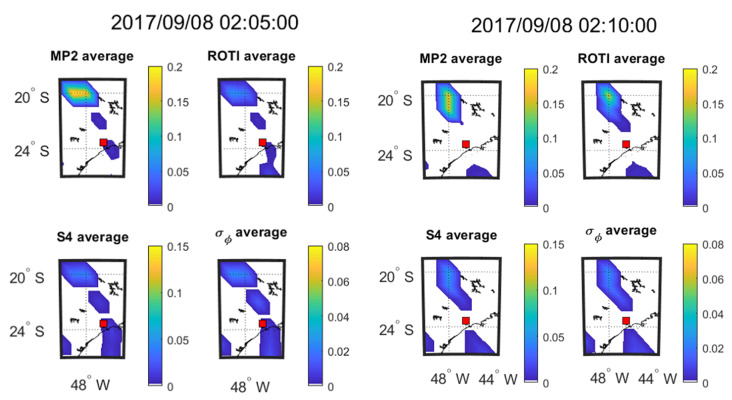
Consecutive 5 min means over 20 min at SAO0P station on 8 September 2017.

**Figure 10:**
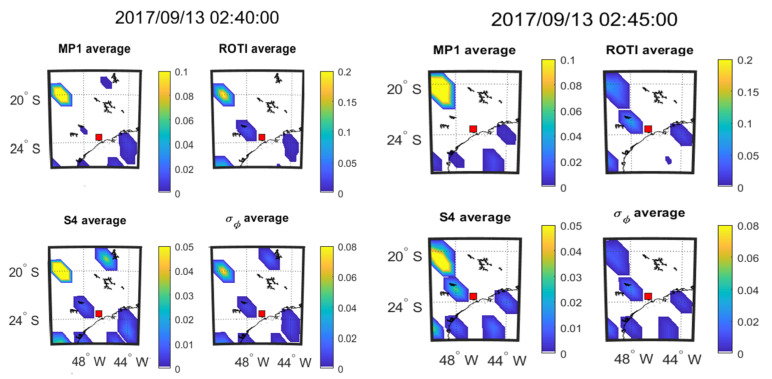

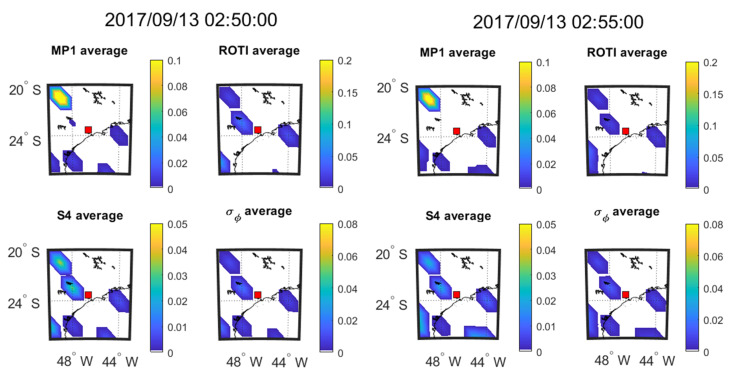
Consecutive 5 min means over 20 min at SAO0P station on 13 September 2017.

The time evolution of the four parameters at every 5 min interval on 08 September 2017 during 01:55–02:10 UT and on 13 September during 02:40–02:55 UT respectively are shown in Figure 9 and Figure 10, which are presented in the form of mean value maps. The SSIM and CC between the parameters in the maps of Figure 9 and Figure 10 are shown respectively in Table 10 and Table 11.

As shown in Figure 9 and Figure 10, mean value maps with 5-min interval are constructed to observe the movements of scintillation and the corresponding change of other parameters. It can be seen from Figure 9 that large value areas (yellow areas) of all the parameters move toward southeast after 20-min propagation. Similarly, it can be observed from Figure 10 that large value areas of all the parameters move southeastward slightly over the 20 min. Therefore, MP, ROTI, S4 and σ_φ_ share the same propagation direction as observed on both the days from Figure 9 and Figure 10. Table 10 shows that the parameters in Figure 9 maintain very high correlations (CC) throughout the 20-min period. The SSIMs fluctuate due to changes in the mean values of the parameters. For Figure 10 and Table 11 shows that ROTI and S4 and ROTI and σ_φ_ maintain a high correlation across the 20-min period. The correlation between MP and S4 declines over the 20-min period. The correlation between MP and σ_φ_ is high in the first 5 min but drops sharply and these two parameters are uncorrelated for the remainder of the period.

### 3.2. Antarctica Data

**Figure 11:**
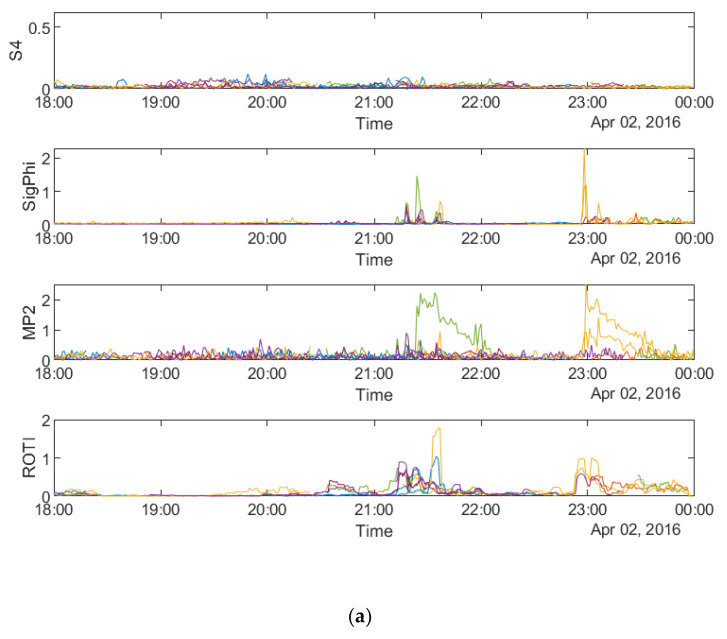

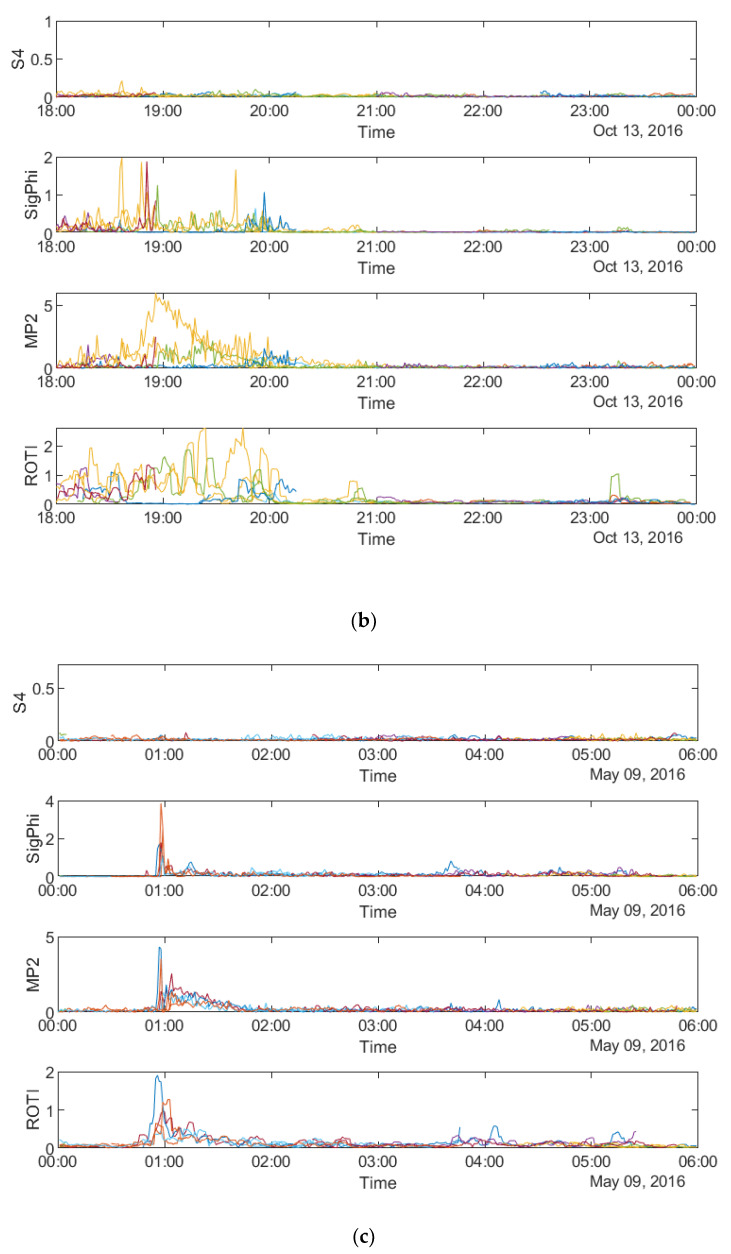
Time series plots of four parameters at SNA0P station: (**a**) during 18:00:00–24:00:00 UTC on 2 April, 2016; (**b**) during 18:00:00–24:00:00 UTC on 13 October, 2016; (**c**) during 00:00:00–06:00:00 UTC on 9 May, 2016.

The time series plots of the four parameters at SNA0P station on 2 April, 13 October 2016 and 9 May 2016 are shown respectively in Figure 11.

**Figure 12:**
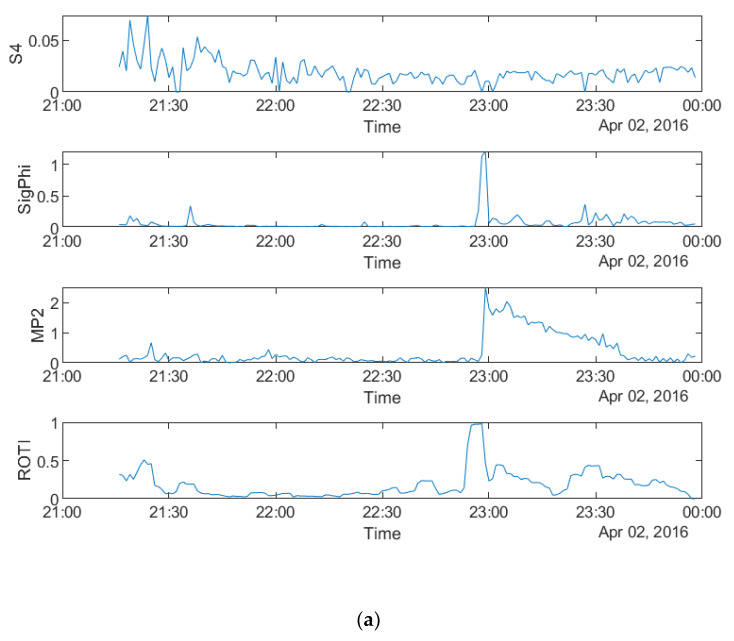

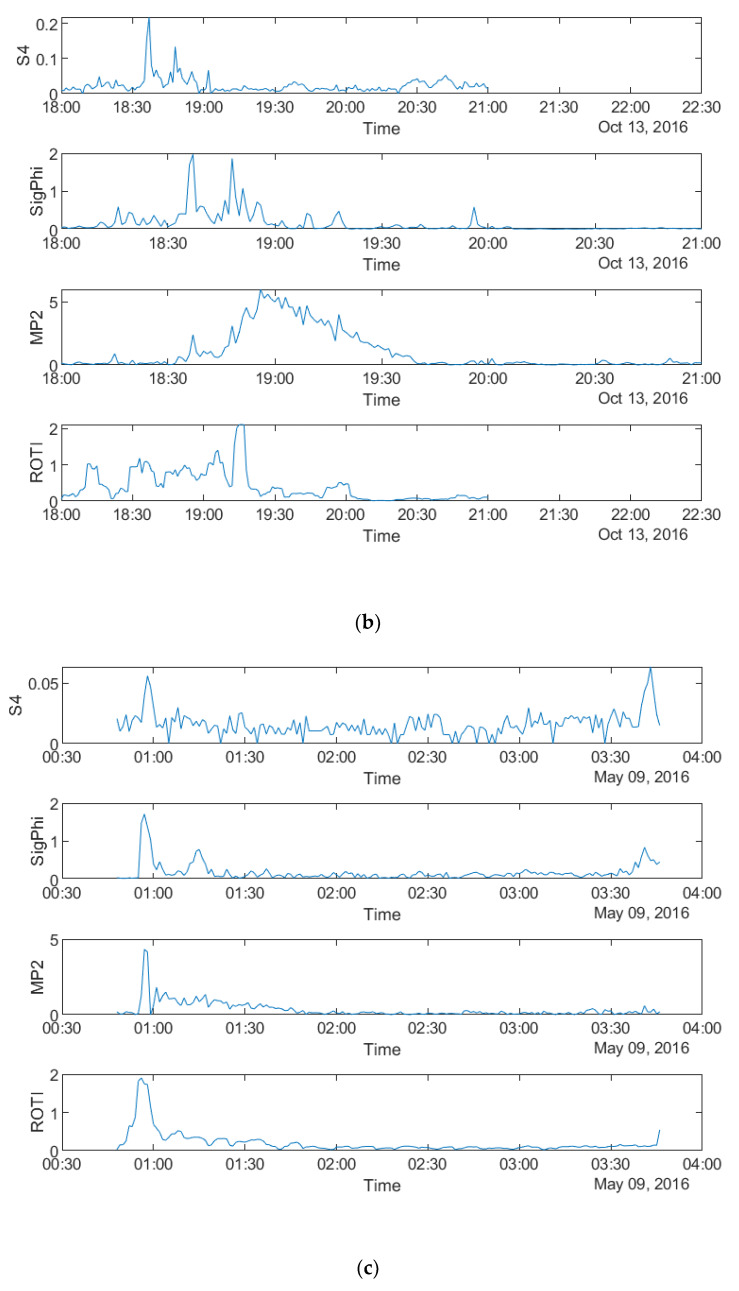
Time series plots of four parameters at SNA0P station on: (**a**) during 18:00:00–24:00:00 UTC on 2 April 2016 for PRN03; (**b**) during 18:00:00–24:00:00 UTC on 13 October 2016 for PRN17; (**c**) during 00:00:00–06:00:00 UTC on 9 May 2016 for PRN08.

As shown in Figure 11, S4 does not have any high values on all three days whereas σ_φ_ still fluctuates in accordance with the feature of scintillation occurrence observed at high latitudes. Therefore, S4 is not considered for comparison in the following paragraphs. Furthermore, it can be seen that all the other three parameters are mainly noisy during the last three hours on 2 April 2016, between 18 and 21 UT on 13 October 2016 and first two hours on 9 May 2016. Different from the data of SAO0P station (Figure 3), the number of satellites affected by scintillation and MP is similar. Figure 12a–c shows the time series plots of the four parameters obtained from a single satellite at SNA0P station respectively on 2 April, 13 October 2016 and 9 May 2016. As shown in Figure 12, a similar high value period can be seen in plots of four parameters on 2 April 2016 and 9 May 2016 though a minor delay exists on 13 October 2016, which supports the temporal relationship between MP, ROTI and σ_φ_.

**Figure 13:**
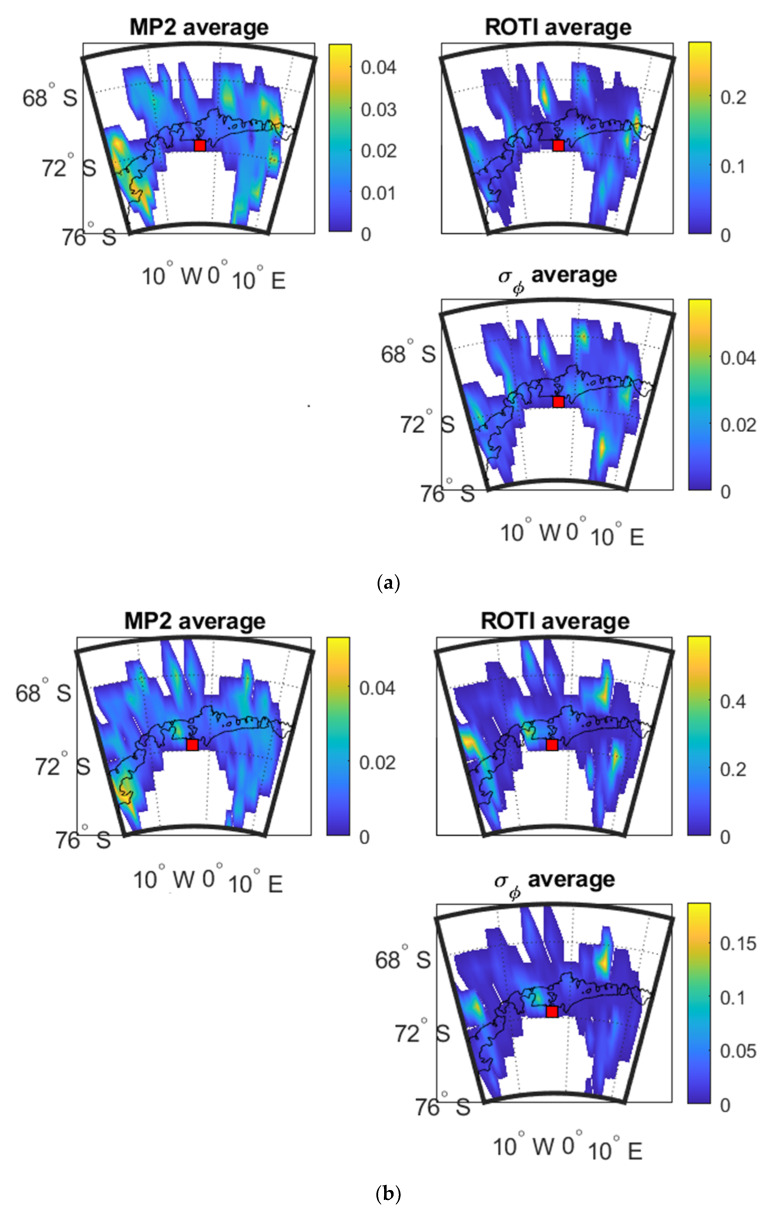

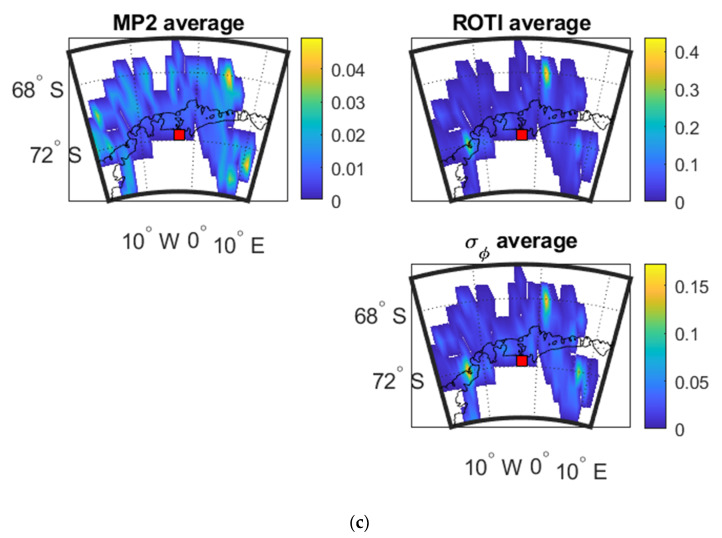
Mean value maps of three parameters at SNA0P station: (**a**) during 18:00:00–24:00:00 UTC on 2 April 2016; (**b**) during 18:00:00–24:00:00 UTC on 13 October 2016; (**c**) during 00:00:00–06:00:00 UTC on 9 May 2016.

Figure 13 shows the mean value maps as a function of IPP latitude and longitude of the three parameters.

As shown in Figure 13, the mean value maps are not as distinct as those in Brazil which can be caused by the sparse distribution of data at the high latitude area. Therefore, the occurrence percentage maps are more useful to this dataset in order to show more details. However, it can still be seen that the high value areas of σ_φ_ maps include those of MP and ROTI maps. The SSIM and CC values between the parameters shown in Figure 13a–c are illustrated in Table 12, Table 13 and Table 14 respectively. As shown in Table 12, Table 13 and Table 14, SSIM values between MP&ROTI and σ_φ_ are all higher than 0.6, where most exceed 0.7. CC values in Table 12, Table 13 and Table 14 show low correlation between MP and σ_φ_, the correlations between ROTI and σ_φ_ are high.

**Figure 14:**
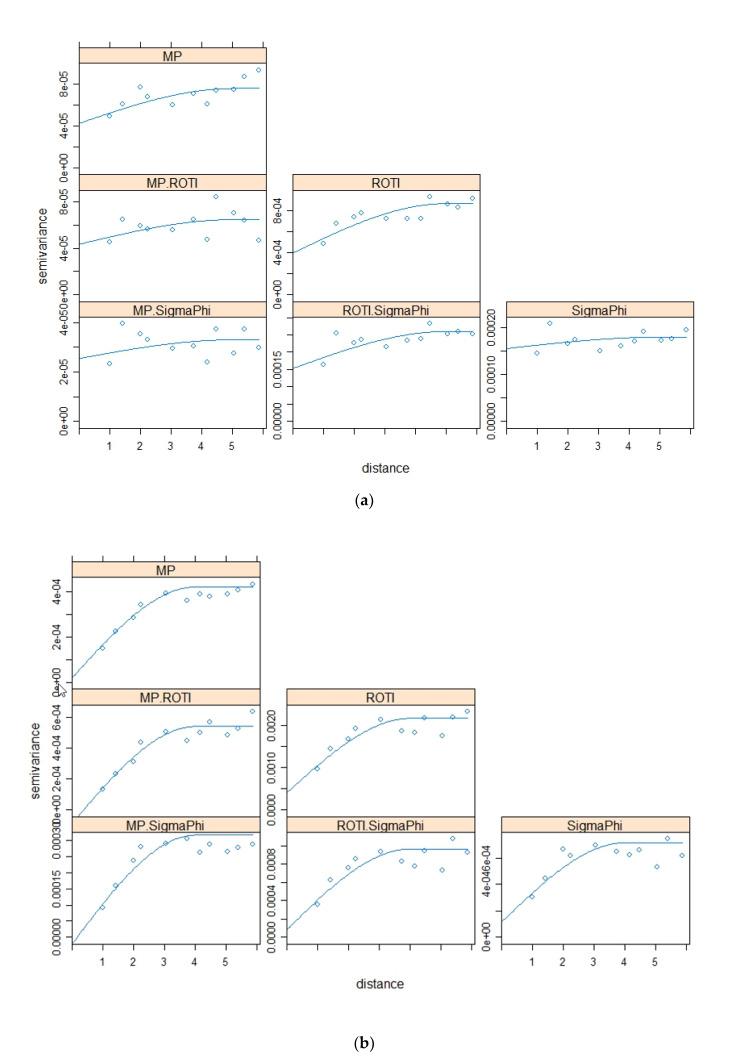

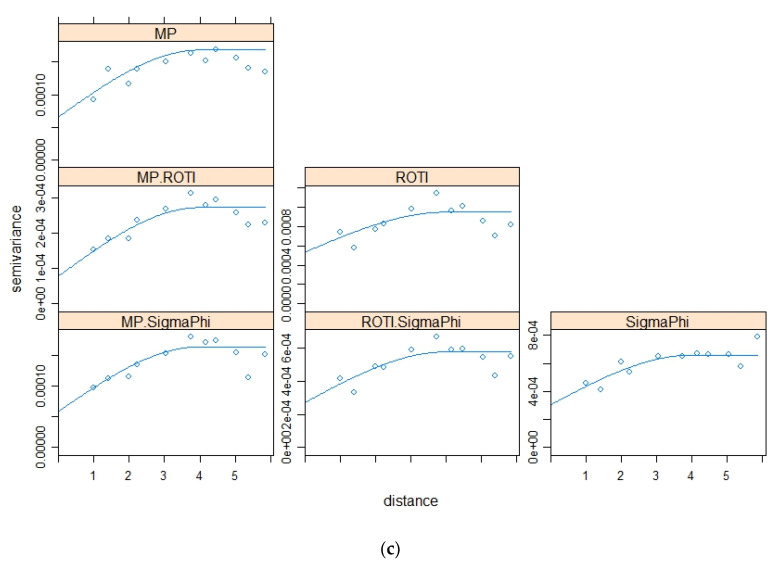
Figure 14a–c shows the variograms and cross-variograms associated with Figure 13a–c.

Figure 14 shows the variograms and cross variograms for the results presented in Figure 13. The variograms and cross-variograms do show evidence of a common spatial structure in the three parameters; however, this is less clear than those for the Brazil data. In particular there is no evidence of spatial correlation for the data from 2016 April 2 (Figure 13a). For 2016 October 13 the variograms and cross-variograms show a clear common spatial structure with a range of approximately 3.5°. For the dataset from 2016 May 9 there is weak evidence of spatial structure.

**Figure 15:**
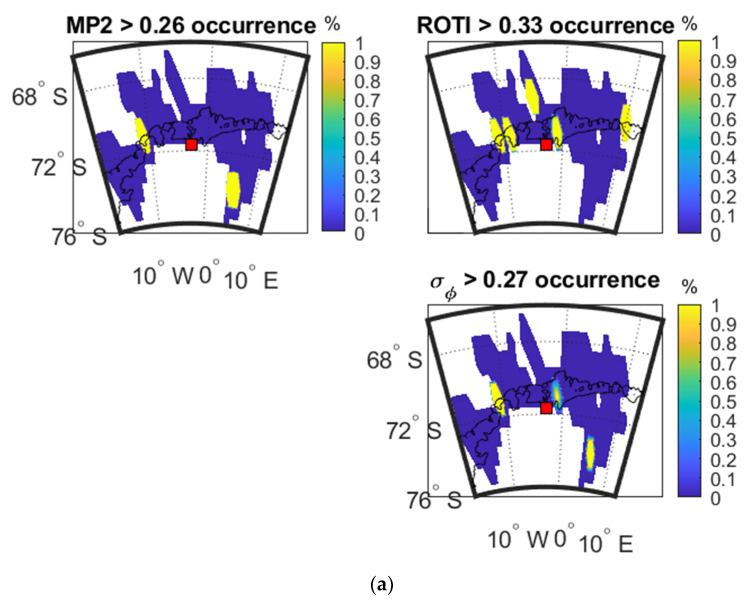

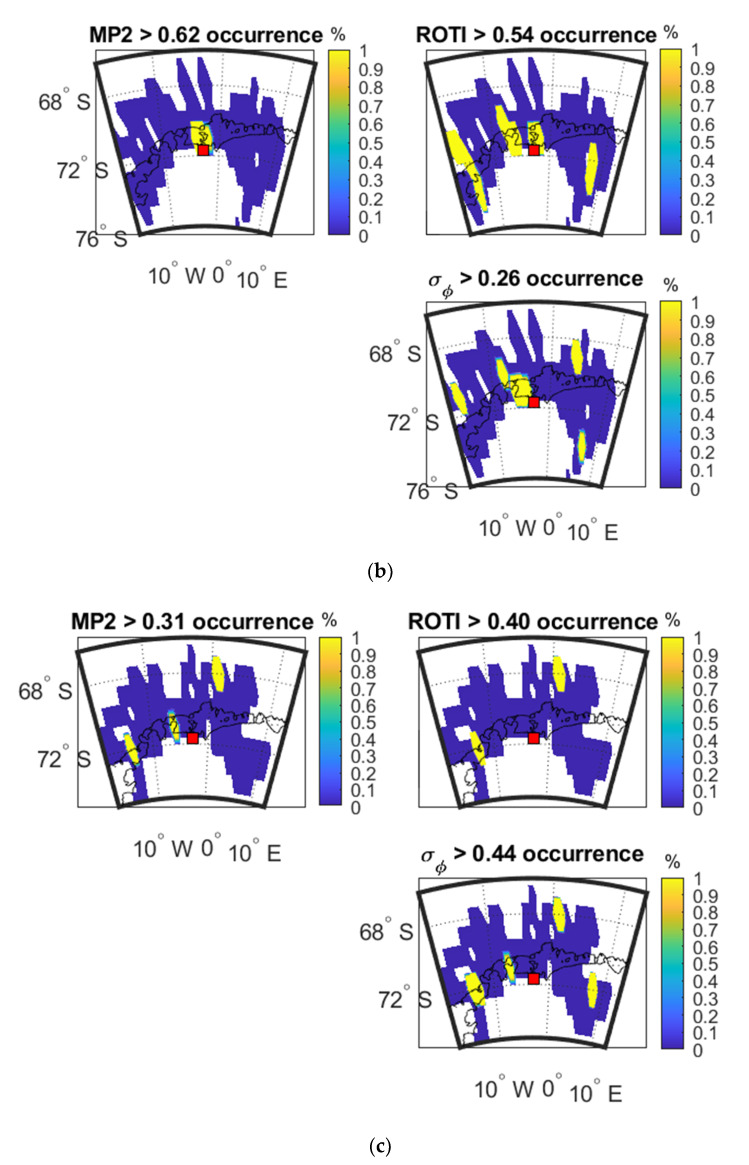
Occurrence percentage maps of three parameters at SNA0P station: (**a**) during 18:00:00–24:00:00 UTC on 2 April 2016; (**b**) during 18:00:00–24:00:00 UTC on 13 October 2016; (**c**) during 00:00:00-06:00:00 UTC on 9 May 2016.

Figure 15 shows the occurrence percentage maps as a function of IPP latitude and longitude for the three parameters respectively on 2 April 2016, 13 October 2016 and 9 May 2016.

As shown in maps of Figure 15a, the high value areas of MP and ROTI separately correspond to different areas of σ_φ_. For instance, some satellites signals less influenced by MP may be more affected by ROTI. Therefore, the combination of two parameters can cover more areas of σ_φ_ than a single parameter. As shown in Figure 15b, high-value areas of ROTI overlap high-value areas of σ_φ_ while MP does not. However, as shown in Figure 15c, MP includes more high-value areas that are spatially-coincident with high-value areas of σ_φ_ than ROTI on 9 May 2016.

Table 15, Table 16 and Table 17 show the SSIM and CC between the three parameters shown in Figure 15a–c.

**Figure 16:**
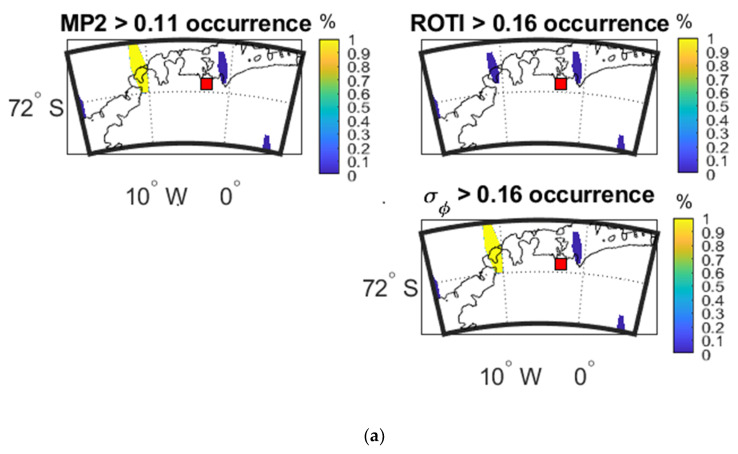

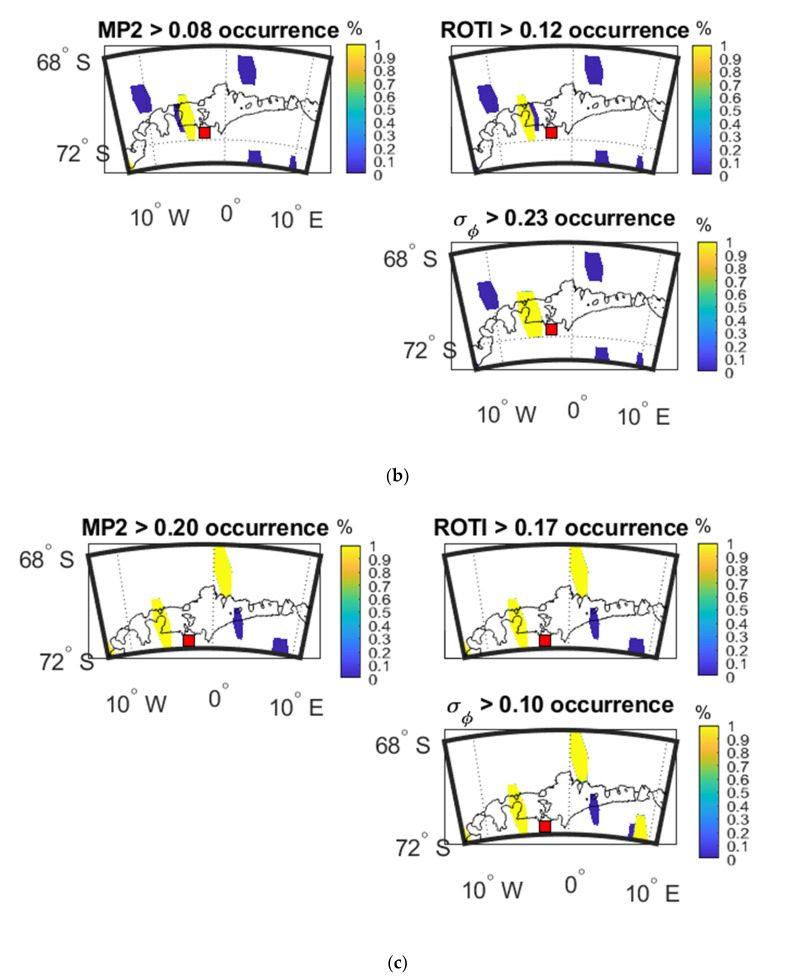
Occurrence percentage maps of three parameters at SNA0P station: (**a**) during 22:59:00–23:04:00 UTC on 2 April 2016; (**b**) during 18:36:00–18:41:00 UTC on 13 October 2016; (**c**) during 01:01:00-01:06:00 UTC on 9 May 2016.

Figure 16 shows the occurrence percentage maps as a function of IPP latitude and longitude of three parameters during the time period when largest variations were observed in Figure 11.

Figure 16a shows that, within a specific time period, MP has the same high-value area as σ_φ_. Figure 16b shows that MP and ROTI both share the high-value areas with σ_φ_. From Figure 16c, both MP and ROTI agree with σ_φ_ for most of the high value areas. These observations are reflected in the CC and SSIM values shown in Table 18, Table 19 and Table 20.

**Figure 17:**
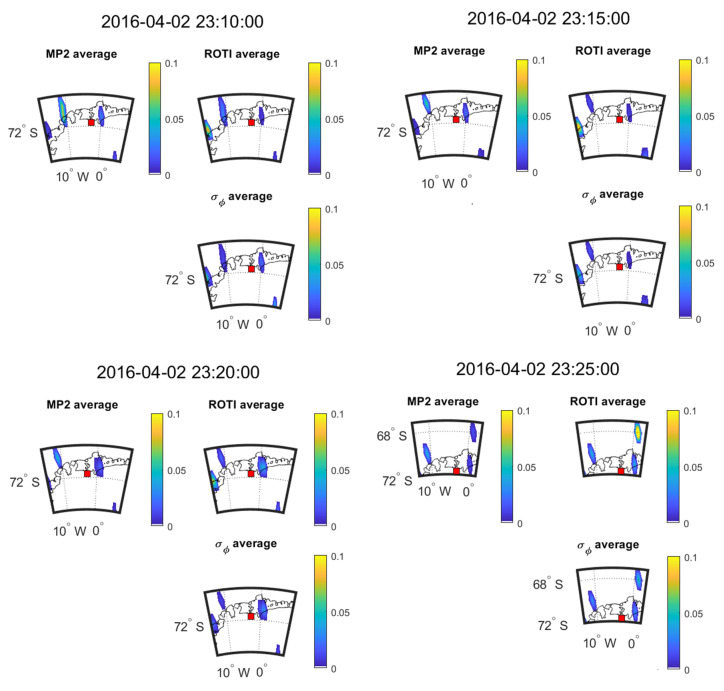
Consecutive 5 min means over 20 min at SNA0P station on 2 April 2016.

**Figure 18:**
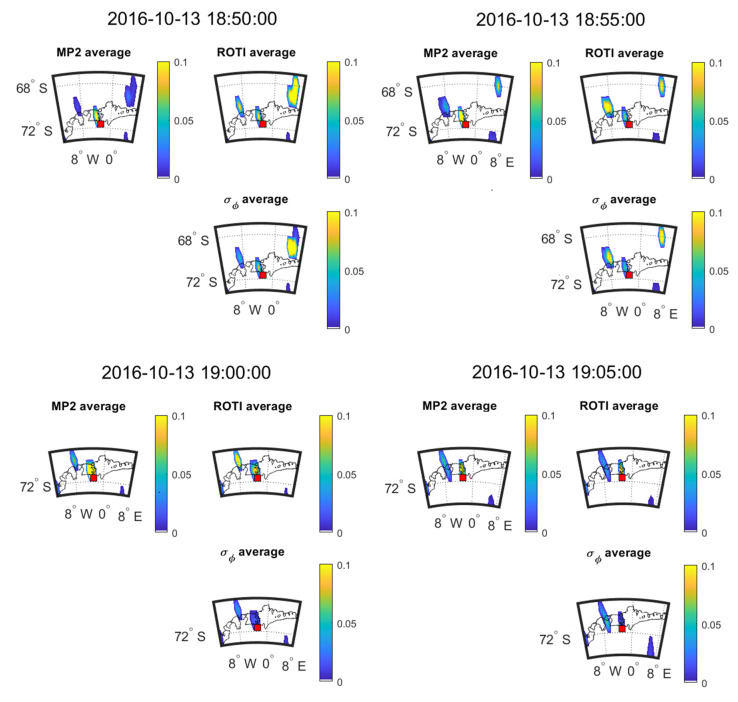
Consecutive 5 min means over 20 min at SNA0P station on 13 October 2016.

Figure 17 and Figure 18 show the time evolution of the three parameters during 23:10–23:25 on 2 April and during 18:50−19:05 on 13 October 2016, respectively. The SSIM and CC between the parameters in the maps of Figure 17 and Figure 18 are shown in Table 21 and Table 22.

Figure 17 and Figure 18 show the change in the 5-min mean values for 2016 April 2 and 2016 October 3 respectively. Figure 17 shows that large value areas of all the parameters move towards northeast across 20 min. However, in Figure 18, the large value areas of parameters move towards the southeast over the 20 min. Again, all the parameters have the same direction for propagation. The results shown in Table 21 and Table 22 are calculated from six and four points, respectively, leading to a high level of uncertainty in the estimation of CC and SSIM. As such we can only make qualitative observations on these plots and cannot back those observations up with reliable CC or SSIM results.

## 4. Discussion

Data obtained from three stations respectively located at equatorial and high-latitude areas are utilized in order to investigate the relationship between MP, ROTI, S4 and σ_φ_. First, as shown in Figure 3, Figure 4, Figure 11 and Figure 12, the relationship in the temporal domain can be observed from all twelve time series plots, where all the parameters show the largest variations during the same time period. Figure 5 and Figure 13 illustrate the spatial relationship for 6-h mean value maps, where high value regions occur at same location on maps. ROTI and σ_φ_ have different high value areas as compared with MP and S4 in Figure 5. This is because the relationship between ROTI and σ_φ_ and between MP and S4 may differ between satellites, due to different effects of ionospheric scintillation. In addition, as shown in Figure 13, high value areas of both MP and ROTI correspond to those of σ_φ_, but relate to different parts of the maps. Hence, it may be possible to identify areas affected by scintillation by combining MP and ROTI maps. It also suggests that MP and ROTI relate to different types of scintillation. This hypothesis can be further evaluated with the occurrence percentage maps. As shown in Figure 7, MP and ROTI separately show similar high value areas as S4 and σ_φ_. As shown in Figure 15, MP and ROTI agree with σ_φ_ over different areas. Next, occurrence percentage maps for 5 min as shown in Figure 8 and Figure 16 suggest similar high value areas for all parameters. Finally, the propagation maps as shown in Figure 9, Figure 10, Figure 17, and Figure 18, where all the parameters move in the similar direction over time, further indicate the spatial similarity between MP&ROTI and S4&σ_φ_.

The Pearson CC and SSIM were both used to quantify the relationship between pairs of maps. CC evaluates the linear correlation whereas SSIM provides a more complete evaluation of map similarity [30]. As discussed in Section 2.7 s(m, n) can be inflated at low values of CC leading to an overoptimistic assessment of the map similarity. For the Brazil data, most of the SSIMs and CCs between S4 and MP exceed those between S4 and ROTI while σ_φ_ correlates more with ROTI than MP. For the Antarctica data, both MP and ROTI sometimes have high similarities with σ_φ._ The variograms and cross-variograms (Figure 6 and Figure 14) were used to illustrate the spatial correlation in the parameters as well the cross-correlation between parameters. The variograms show clear spatial structure, with a range of 5 to 6°, for the Brazil data. Likewise, the cross-variograms show a common spatial structure. Whereas CC quantifies the bi-variate correlation the cross-variograms quantifies whether the spatial structure is common between the different parameters. This gives evidence of strong spatial correlation between the four parameters and backs-up the results observed for the SSIM and CC. For the Antarctica case study there is clear evidence of spatial correlation for the dataset from 2016-10-13, with a common range of approximately 3.5°. There is less clear evidence for the dataset from 2016-05-09 (common range of approximately 3.5°) and no evidence of spatial correlation for the dataset from 2016-04-02

The purpose of this paper is to explore the relationship between MP, ROTI and scintillation parameters. We aim to better understand the relationships first, leaving open the possibility to use these relationships to provide supplementary information that may assist in overcoming the limitations of using specialized ionospheric scintillation monitoring receivers. The relationship between MP and scintillation has been investigated by Romano et al. [8] and shows that the presence of obstacles in the vicinity of receivers can lead to the increase of S4 that shows some agreement with areas of MP measured using code-carrier divergence (CCD) standard deviation. Romano et al. [8] also shows that a high percentage of the higher S4 values and CCD are below 30°. In this study, all data has a 30° satellite mask applied which should significantly reduce any MP effect in the vicinity of the station. Therefore, the high values of S4, σ_φ_ and MP in this study all occur for satellites at high elevations (>30°), giving support to the theory that the higher values of MP are being influenced by the scintillation events evidenced from the high scintillation indices values during the corresponding time window.

The relationship between MP values from the TEQC software and σ_φ_ was observed by Hancock et al. [10] through occurrence number plots and time series plots from data collected in Hong Kong, which provided initial evidence that the study of the relationship between MP and scintillation parameters may be interesting and of possible use in the mitigation of errors during scintillation events. Following on Romano et al. [8] and Hancock et al. [10] this study provides a much deeper statistical analysis of the relationships between MP, S4 and σ_φ_. Furthermore, the ionospheric index ROTI is added in this study to give additional evidence that scintillation is real rather than a product of the physical environment around the receiver as claimed by Romano et al. [8].

The relationship between ROTI and scintillation has been investigated [9,11,31,32,33]. The scatter plot and the time series plot are common methods used in recent studies to demonstrate the linear relationship between ROTI, S4 and σ_φ_ [9,11,31,32]. Additionally, the relationship between ROTI and scintillation has been shown to be affected by changes in elevation angle [11]. Correlation coefficients have shown distinctly higher values when the satellite elevation angle exceeds 60° when compared to satellites with elevation angles lower than 60°. However, this investigation focuses on elevations higher than 30°, focusing on satellites with higher elevation angles thus taking advantage of the stronger relationship between scintillation and ROTI shown by Yang and Liu [11]. Analysis of the relationship between MP and the scintillation parameters with respect to the effects of elevation angle are not part of this study.

Acharya and Majumdar [33], used statistical analysis to conclude that the probability density distribution of S4 can be obtained using ROTI, thereafter, the occurrence probability of scintillation can be estimated. Therefore, Acharya and Majumdar [33] gives the conclusion about strong evidence of a relationship between ROTI and S4, which in general agrees with the analysis from this study. In addition, Carrano et al. [31] also demonstrated the theoretical relationship between ROTI and S4 and has demonstrated that this relationship is highly dependent on the sampling rate. They also provide reasons why this relationship varies between different dates and in different regions. These factors have not been investigated in this study and further work is required in this area. Therefore, the investigation on the relationship based on first principles and on the formulae between MP and scintillation is required in future work.

Investigations [9,31,32,33] focus on S4, where σ_φ_ was not investigated. Therefore, the investigation on σ_φ_ is less thorough than that on S4 based on the past research [9,31,32,33]. Though σ_φ_ was investigated by Yang and Liu [11], a comparison of how both scintillation indices are related to ROTI was not undertaken. According to the graphs given by Yang and Liu [11], S4 is more correlated with ROTI than σ_φ_, which is caused by the relative inactivity of σ_φ_ (σ_φ_ shows a maximum value less than 0.4). In contrast, both S4 and σ_φ_ are analyzed and compared in this paper with reference to their relationships with ROTI. Furthermore, analysis from the stations in this study show evidence that ROTI is more similar to σ_φ_ than to S4 both visually and statistically, giving new valuable insights into the relationships between ROTI, S4 and σ_φ_.

Previous research has focused principally on the linear relationship between ROTI and the scintillation parameters, this study adds to this body of research by investigating the spatial relationship. This leads to the possibility of using these data sets to generate scintillation risk maps, that may be similar in principle to tracking jitter maps generated Sreeja et al. [34], and also similar to Figure 4, Figure 5, Figure 6 and Figure 7 given by Koulour et al. in [35], which visualize the effect of scintillation and can be used to identify and possibly mitigate risk caused by scintillation events. Figure 2 from Sreeja et al. in [34] shows the S4 maps as a function of time and IPP latitude where strong scintillation activity was observed between 18° S and 26° S from 8pm to 0am local time on March 9–11, 2011. By comparison, Figure 3c, Figure 5c and Figure 7c in our paper show all the parameters are intense between 18° S and 26° S from 0 am to 4 am UTC (8 pm to 0 am in local time) on March 12 2011, which is similar to the output obtained by Sreeja et al. [34].

Additionally, ROTI is the major proxy to the scintillation parameters proposed in previous research, which has shown weaknesses in the ability of ROTI to replace S4 and σ_φ_. In this research the relationship between ROTI and the scintillation parameters S4 and σ_φ_ has been further investigated and in addition this study shows that it may be possible to add MP as additional parameter, in support of other parameters such as ROTI, computed from a standard GNSS receiver (i.e., non-scintillation monitoring receiver) that may be indicative of scintillation. However, the increase of MP does not generally indicate the occurrence of scintillation events as the scintillation measurements can be contaminated by real multipath effects [8].

## 5. Conclusions

Six days of data under strong scintillation from three stations, respectively located in Brazil and Antarctica, are utilized to research on the relationship between MP and ROTI and scintillation indices. The relationship in the temporal and spatial domain is evaluated with a series of data science techniques, including 2D map comparison, Pearson CC, SSIM and variograms. The propagation patterns of all parameters are demonstrated to be similar. According to the discussion, it can be concluded that:

(1) A relationship between MP, ROTI and scintillation exists in both temporal and spatial domain
(2) An integration between MP and ROTI can more completely represent scintillation than a single parameter. Precisely, MP and ROTI can reflect different types of scintillation. For equatorial data, MP is more correlated with S4 while ROTI is more relevant to σ_φ_. For high latitude data, it is possible for both MP and ROTI to be correlated with σ_φ_.
(3) The propagation patterns of MP, ROTI and S4, σ_φ_ are similar, which can contribute to the prediction of scintillation with MP and ROTI.
(4) MP is correlated with scintillation only when scintillation is active while ROTI correlates with scintillation during both quiet and intense periods.

In the future, a broader network with more data should be utilized to investigate the relationship with more comprehensive characteristics of MP, ROTI, S4 and σ_φ_. Furthermore, more detailed studies on the possibility of separating the effects of real multipath and the effects of scintillation in MP data is needed that could include detailed station characterizations.

## Figures and Tables

**Table 1:** SSIM and CC between the maps shown in Figure 5a.

	MP		ROTI	
Criteria	SSIM	CC (*p*-value)	SSIM	CC (*p*-value)
S4	0.75	0.81 (<0.01)	0.73	0.76 (<0.01)
σ_φ_	0.67	0.74 (<0.01)	0.79	0.88 (<0.01)

**Table 2:** SSIM and CC between the maps shown in Figure 5b.

	MP		ROTI	
Criteria	SSIM	CC (*p*-value)	SSIM	CC (*p*-value)
S4	0.73	0.71(<0.01)	0.70	0.68 (<0.01)
σ_φ_	0.65	0.58 (<0.01))	0.80	0.93 (<0.01)

**Table 3:** SSIM and CC between the maps shown in Figure 5c.

	MP		ROTI	
Criteria	SSIM	CC (*p*-value)	SSIM	CC (*p*-value)
S4	0.73	0.70 (<0.01)	0.63	0.77 (<0.01)
σ_φ_	0.56	0.43 (<0.01)	0.80	0.66 (<0.01)

**Table 4:** SSIM and CC between the maps shown in Figure 7a.

	MP		ROTI	
Criteria	SSIM	CC (*p*-value)	SSIM	CC (*p*-value)
S4	0.71	0.52 (<0.01)	0.75	0.02 (0.87)
σ_φ_	0.58	−0.03 (0.80)	0.79	0.83 (<0.01)

**Table 5:** SSIM and CC between the maps shown in Figure 7b.

	MP		ROTI	
Criteria	SSIM	CC (*p*-value)	SSIM	CC (*p*-value)
S4	0.83	0.77 (<0.01)	0.70	0.66 (<0.01)
σ_φ_	0.69	0.56 (<0.01)	0.71	0.98(<0.01)

**Table 6:** SSIM and CC between the maps shown in Figure 7c.

	MP		ROTI	
Criteria	SSIM	CC (*p*-value)	SSIM	CC (*p*-value)
S4	0.64	−0.04 (0.77)	0.61	−0.04 (0.73)
σ_φ_	0.63	−0.03 (0.79)	0.76	0.72 (<0.01)

**Table 7:** SSIM and CC between the maps shown in Figure 8a.

	MP		ROTI	
Criteria	SSIM	CC (*p*-value)	SSIM	CC (*p*-value)
S4	0.96	1.00 (<0.01)	0.96	1.00 (<0.01)
σ_φ_	0.59	1.00 (<0.01)	0.59	1.00 (<0.01)

**Table 8:** SSIM and CC between the maps shown in Figure 8b.

	MP		ROTI	
Criteria	SSIM	CC (*p*-value)	SSIM	CC (*p*-value)
S4	0.96	1.00 (<0.01)	0.96	1.00 (<0.01)
σ_φ_	0.83	1.00 (<0.01)	0.83	1.00 (<0.01)

**Table 9:** SSIM and CC between the maps shown in Figure 8c.

	MP		ROTI	
Criteria	SSIM	CC (*p*-value)	SSIM	CC (*p*-value)
S4	1.00	1.00 (<0.01)	1.00	1.00 (<0.01)
σ_φ_	1.00	1.00 (<0.01)	1.00	1.00 (<0.01)

**Table 10:** SSIM and CC between the maps shown in Figure 9.

8 September 2017	01:55:00		02:00:00		02:05:00		02:10:00	
Criteria	SSIM	CC (*p*-Value)	SSIM	CC (*p*-Value)	SSIM	CC (*p*-Value)	SSIM	CC (*p*-Value)
MP vs. S4	0.85	0.98 (< 0.01)	0.39	0.99 (< 0.01)	0.38	0.99 (< 0.01)	0.50	0.98 (< 0.01)
MP vs. σ_φ_	0.59	1.00 (< 0.01)	0.33	1.00 (< 0.01)	0.27	0.94 (< 0.01)	0.31	0.97 (< 0.01)
ROTI vs. S4	0.88	0.88 (< 0.01)	0.78	0.97 (< 0.01)	0.89	0.93 (< 0.01)	0.64	0.93 (< 0.01)
ROTI vs. σ_φ_	0.75	0.97 (< 0.01)	0.73	0.98 (< 0.01)	0.76	0.93 (< 0.01)	0.40	0.93 (< 0.01)

**Table 11:** SSIM and CC between the maps shown in Figure 10.

13 September 2017	02:40:00		02:45:00		02:50:00		02:55:00	
Criteria	SSIM	CC (*p*-Value)	SSIM	CC (*p*-Value)	SSIM	CC (*p*-Value)	SSIM	CC (*p*-Value)
MP vs. S4	0.84	0.95 (<0.01)	0.46	0.71 (0.03)	0.44	0.57 (0.24)	0.45	0.24 (0.57)
MP vs. σ_φ_	0.73	0.96 (<0.01)	0.26	−0.09 (0.82)	0.28	−0.20 (0.71)	0.43	−0.11 (0.80)
ROTI vs. S4	0.74	0.88 (<0.01)	0.68	0.63 (0.07)	0.64	0.62 (0.19)	0.86	0.63 (0.09)
ROTI vs. σ_φ_	0.60	0.93 (<0.01)	0.49	0.82 (<0.01)	0.51	0.93 (<0.01)	0.82	0.90 (<0.01)

**Table 12:** SSIM and CC between the maps shown in Figure 13a.

	MP		ROTI	
Criteria	SSIM	CC (*p*-value)	SSIM	CC (*p*-value)
σ_φ_	0.73	0.23 (0.01)	0.77	0.65 (<0.01)

**Table 13:** SSIM and CC between the maps shown in Figure 13b.

	MP		ROTI	
Criteria	SSIM	CC (*p*-value)	SSIM	CC (*p*-value)
σ_φ_	0.78	0.54 (<0.01)	0.69	0.76 (<0.01)

**Table 14:** SSIM and CC between the maps shown in Figure 13c.

	MP		ROTI	
Criteria	SSIM	CC (*p*-value)	SSIM	CC (*p*-value)
σ_φ_	0.61	0.42 (<0.01)	0.79	0.69 (<0.01)

**Table 15:** SSIM and CC between the maps shown in Figure 15a.

	MP		ROTI	
Criteria	SSIM	CC (*p*-value)	SSIM	CC (*p*-value)
σ_φ_	0.82	0.46 (<0.01)	0.65	0.03 (0.74)

**Table 16:** SSIM and CC between the maps shown in Figure 15b.

	MP		ROTI	
Criteria	SSIM	CC (*p*-value)	SSIM	CC (*p*-value)
σ_φ_	0.71	0.41 (<0.01)	0.46	0.38 (<0.01)

**Table 17:** SSIM and CC between the maps shown in Figure 15c.

	MP		ROTI	
Criteria	SSIM	CC (*p*-value)	SSIM	CC (*p*-value)
σ_φ_	0.86	0.45 (<0.01)	0.79	0.66 (<0.01)

**Table 18:** SSIM and CC between the maps shown in Figure 16a.

	MP		ROTI	
Criteria	SSIM	CC (*p*-value)	SSIM	CC (*p*-value)
σ_φ_	1.00	1.00 (<0.01)	0.01	0.00 (NaN)

**Table 19:** SSIM and CC between the maps shown in Figure 16b.

	MP		ROTI	
Criteria	SSIM	CC (*p*-value)	SSIM	CC (*p*-value)
σ_φ_	0.17	−0.08 (0.83)	0.90	0.97 (<0.01)

**Table 20:** SSIM and CC between the maps shown in Figure 16c.

	MP		ROTI	
Criteria	SSIM	CC (*p*-value)	SSIM	CC (*p*-value)
σ_φ_	0.44	0.72 (0.11)	0.63	0.92 (0.01)

**Table 21:** SSIM and CC between the maps shown in Figure 17.

2 April 2016	23:10:00		23:15:00		23:20:00		23:25:00	
Criteria	SSIM	CC (*p*-Value)	SSIM	CC (*p*-Value)	SSIM	CC (*p*-Value)	SSIM	CC (*p*-Value)
MP vs. σ_φ_	0.02	−0.63 (0.18)	0.47	−0.33 (0.53)	0.70	−0.29 (0.58)	0.31	−0.50 (0.50)
ROTI vs. σ_φ_	0.47	0.26 (0.62)	0.60	0.80 (0.06)	0.62	0.38 (0.45)	0.50	0.37 (0.63)

**Table 22:** SSIM and CC between the maps shown in Figure 18.

13 October 2016	18:50:00		18:55:00		19:00:00		19:05:00	
Criteria	SSIM	CC (*p*-Value)	SSIM	CC (*p*-Value)	SSIM	CC (*p*-Value)	SSIM	CC (*p*-Value)
MP vs. σ_φ_	0.07	−0.00 (0.99)	0.36	0.32 (0.48)	0.14	0.08 (0.88)	0.31	0.25 (0.58)
ROTI vs. σ_φ_	0.52	0.47 (0.29)	0.80	0.79 (0.03)	0.30	0.79 (0.06)	0.38	0.31 (0.50)
